# Opinions of speech-language-hearing pathologists and students on evidence-based practice: a systematic review

**DOI:** 10.1590/2317-1782/e20250300en

**Published:** 2026-07-06

**Authors:** Samara Fernandes da Silva Souza, Allya Francisca Marques Borges, Ramon Cipriano Pacheco de Araújo, Denise da Silva Medeiros, Suzanne Bettega Almeida, Cristiano Miranda de Araujo, Hipólito Virgílio Magalhães, Karinna Veríssimo Meira Taveira

**Affiliations:** 1 Departamento de Fonoaudiologia, Universidade Federal do Rio Grande do Norte – UFRN - Natal (RN), Brasil.; 2 Grupo de Pesquisa Estudos em Motricidade Orofacial e Disfagia Orofaríngea, Universidade Federal do Rio Grande do Norte – UFRN - Natal (RN), Brasil.; 3 Programa de Pós-graduação em Saúde da Comunicação Humana, Universidade Federal de Pernambuco – UFPE – Recife (PE), Brasil.; 4 Núcleo de Estudos Avançados em Revisão Sistemática e Meta-análise – NARSM, Programa de Pós-graduação em Distúrbios da Comunicação, Universidade Tuiuti do Paraná – UTP - Curitiba (PR), Brasil.; 5 Programa de Pós-graduação em Odontologia, Pontifícia Universidade Católica do Paraná - PUC - Curitiba (PR), Brasil.; 6 Núcleo de Estudos Avançados em Revisão Sistemática e Meta-análise – NARSM, Programa Associado de Pós-graduação em Fonoaudiologia, Departamento de Morfologia, Centro de Biociências, Universidade Federal do Rio Grande do Norte – UFRN - Natal (RN), Brasil.

**Keywords:** Evidence-Based Practice, Speech-Language Pathology, Attitude, Knowledge, Systematic Review

## Abstract

**Purpose:**

To synthesize evidence on the knowledge, skills, attitudes, behaviors, and barriers reported by speech-language-hearing pathologists and students regarding evidence-based practice (EBP).

**Research strategy:**

This systematic review searches the PubMed/MEDLINE, Scopus, Web of Science, Embase, LILACS, SciELO, and LIVIVO databases and grey literature.

**Selection criteria:**

The review included observational studies that investigated aspects related to EBP among speech-language-hearing professionals and undergraduates, with data collected through questionnaires.

**Data analysis:**

Two reviewers extracted data independently, and information synthesis was supported by NotebookLM artificial intelligence, with subsequent manual verification. The risk of bias was assessed considering sample representativeness, response rate, data precision, evidence of sample size calculation, and quality of the instrument used.

**Results:**

31 studies published between 2004 and 2024 were included, with samples ranging from 9 to 2,762 participants. The risk of bias ranged from 1 to 4 on a scale of 0 to 6. Studies show generally favorable attitudes towards EBP, but indicate important limitations in database search skills, critical reading, and application of evidence. Clinical practice is still heavily based on personal experience, with limited use of scientific literature. The main barriers reported were lack of time, limited access to evidence, scarcity of applicable evidence, and gaps in training.

**Conclusion:**

Despite positive attitudes, the adoption of EBP in speech-language-hearing pathology is still limited, requiring educational and institutional strategies to strengthen its implementation.

## INTRODUCTION

Evidence-based practice (EBP) refers to a set of criteria used to evaluate scientific evidence. Several health fields, including medicine, physiotherapy, nursing, speech-language-hearing (SLH) therapy, occupational therapy, nutrition, and dentistry, have adopted EBP^(1-5)^. Its goal is to use the best available research evidence to inform clinical healthcare decisions, ensuring that patients receive up-to-date, safe, and effective treatments. EBP is the integration of three components: the professional's clinical experience, external and internal evidence, and the perspectives of the client and/or patient and caregiver^(6)^. In this sense, the implementation of EBP is an important clinical resource for SLH therapy, because, besides discussing and implementing existing evidence, it constantly reflects on the needs of the area and the insufficiency of evidence to support some clinical practice management strategies, regarding the quality and quantity of evidence^(7)^.

Although the concepts of EBP are well defined, its implementation faces several challenges^(8)^. The latter are closely related to the current state of health policies, the complexity of SLH practice, and access to studies and continuing education programs. Previous studies with different health professions have identified several barriers, such as the lack of time^(9-12)^, lack of access to full-text articles^(13,14)^, and lack of skills to find and understand studies^(11,14-16)^. The inability of health professionals to understand and select high-quality studies has been attributed to inadequate training in EBP, due to the wide variability of teaching methods during university education^(16)^. Other barriers include the questionable quality of studies^(15,17,18)^ and conflicting results from different studies on the same topic^(10,18)^, and a lack of mastery of search strategies and critical appraisal of scientific articles^(1)^. Some studies present characteristics that do not represent real clinical practice, hindering their application^(11,19,20)^. Another factor that can interfere with the application of EBP is the language of publication, since most studies are published in English^(21)^, which can make it difficult for readers who are not proficient in that language to use it.

Despite having some theoretical basis, SLH pathologists who work with language disorders and other areas of SLH pathology internationally recognize insufficient time, extensive workload, scarcity of research in the area, quality of available evidence, and lack of resources in the work environment as the main obstacles to executing EBP^(2,22-24)^. Formal training on the basics of EBP during undergraduate studies or continuing education appears as a strong predictor for using EBP in clinical practice^(22,25,26)^.

Therefore, it is important to conduct a systematic review to better inform professionals about these characteristics and identify the most relevant difficulties faced by SLH pathologists regarding EBP. Thus, this study aimed to systematically review the evidence on the knowledge, skills, behaviors, opinions, and barriers faced by SLH pathologists concerning EBP.

## METHODS

### Protocol and Registry

The protocol for this systematic review was registered in PROSPERO^®^ (CRD42025647859) (International Prospective Register of Systematic Reviews - Centre for Reviews and Dissemination, University of York) and conducted in accordance with the PRISMA-2020 checklist (Preferred Reporting Items for Systematic Reviews and Meta-Analyses)^(27)^.

### Research Question

**Figure 1:**
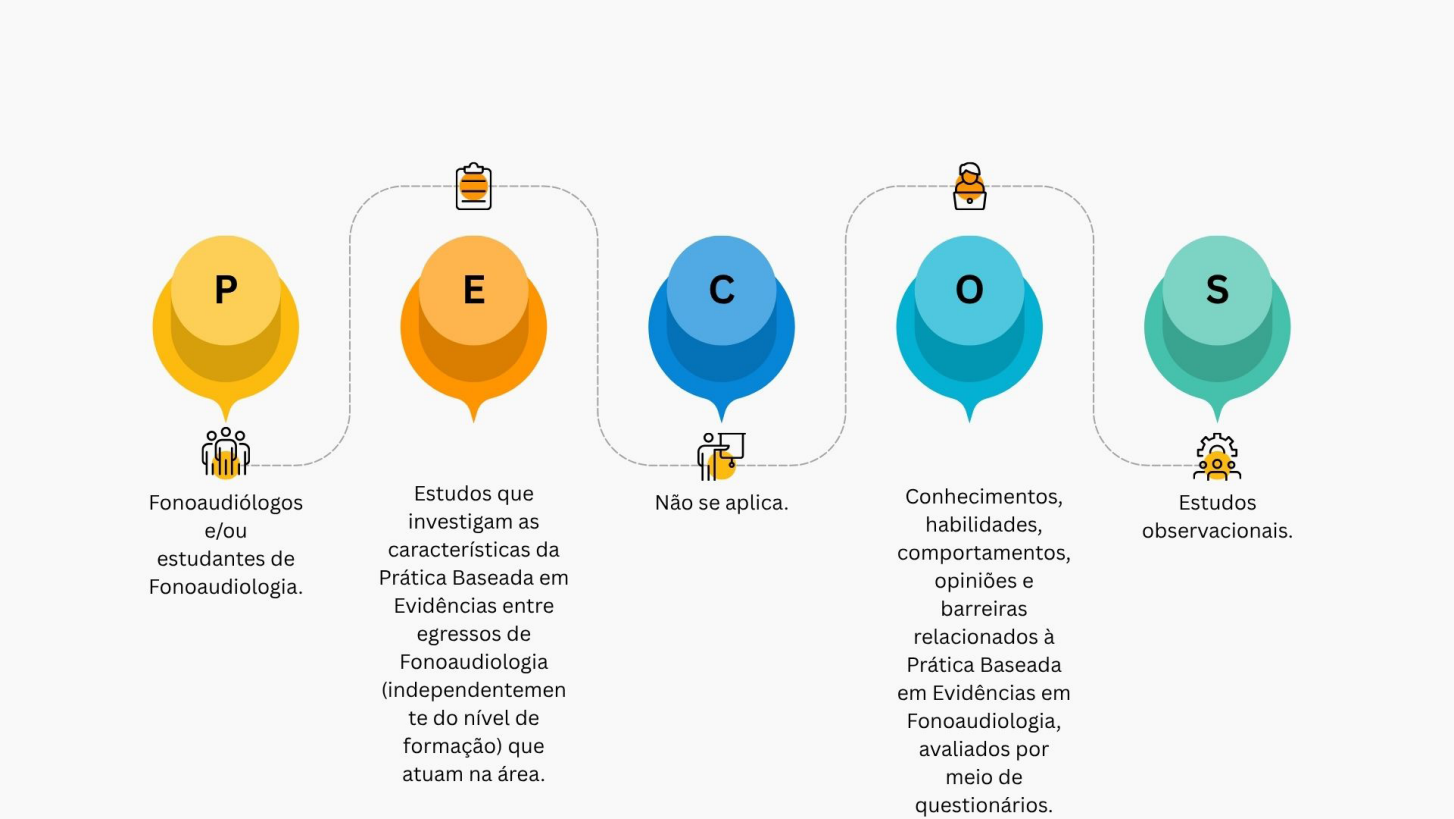
The acronym “PECOS” as a guide to identifying the research question

The PECOS acronym (Participants, Exposure, Control, Outcomes, and Study Design) guided the construction of the research question. Figure 1 describes the structure of PECOS used to address the following focused question: “What knowledge, skills, behaviors, opinions, and barriers do SLH pathologists face in relation to EBP?”.

### Eligibility Criteria

The review included observational cohort and cross-sectional studies that investigated the characteristics of EBP among SLH pathologists and undergraduate students, focusing on knowledge, skills, behaviors, opinions, and barriers, assessed through questionnaires. Studies were not excluded based on language, sex, or publication date.

The review excluded:

1. Studies that did not involve SLH students or professionals.
2. Studies that did not report knowledge, skills, behavior, opinions, or barriers in SLH pathology related to EBP.
3. Reviews, letters to the editor, case reports, case series, expert opinions, and guidelines.

### Information sources and search strategy

Word truncations and combinations were adapted for each search strategy in the following databases: Embase, Latin American and Caribbean Health Sciences Literature (LILACS), LIVIVO, PubMed (including MEDLINE), Scientific Electronic Library Online (SciELO), Scopus, and Web of Science. Descriptors, keywords, and free terms related to "Evidence-Based Practice," "Attitude," "Opinions," "Knowledge," "Barrier," "Ability," and "Speech-Language Pathologists" were used, combined with Boolean operators (OR and AND). The complete search was conducted on November 19, 2024, and can be consulted in Appendix 1. The grey literature was searched via Google Scholar and ProQuest Dissertations & Theses. Manual searches were also conducted in the reference lists of the included studies, and experts were consulted to identify additional studies. EndNote^®^ X7 (Thomson Reuters, Philadelphia, PA) was used to manage references and eliminate duplicates. The Rayyan^®^ platform was used for the selection of studies, with blind review by two reviewers and the participation of a third reviewer as moderator.

### Selection of studies

Two review authors (SFSS and DSM) independently screened titles and abstracts identified by the search strategy based on eligibility criteria (Phase 1). They then independently assessed the full texts of the studies, considering the inclusion and exclusion criteria (Phase 2). Disagreements were resolved through discussion with a third author and subsequent consensus among the three reviewers (KVMT).

### Data collection and data list

Data were extracted using NotebookLM artificial intelligence^(28)^, which assisted in reading, categorizing, and synthesizing the included scientific texts. The following information was extracted from each study: author, year of publication, country of study, sample characteristics, number of participants (including response rate, when reported), instrument used, and main aspects of EBP investigated. The information processed by the tool was verified by one of the reviewers (SFSS), and any doubts or discrepancies were discussed and resolved jointly with a second reviewer (KVMT) with prior experience in the subject matter of the review.

### Risk of bias in each study

The risk of bias was assessed using criteria previously described in the literature^(29,30)^, which consider sample representativeness, response rate, data precision, presentation of sample power calculation, and the instrument used. Each study received a score on a scale of 0 to 6 points, where higher scores indicate a lower risk of bias. Two reviewers (SFSS and DSM) conducted the assessment independently, and any discrepancies were resolved with the mediation of a third reviewer (KVMT).

### Assessment of reporting bias

Given the impossibility of assessing publication bias through graphical analysis (n < 10), a broad search strategy was carried out across various databases and grey literature, including a database in a language other than English.

## RESULTS

### Selection of studies

**Figure 2:**
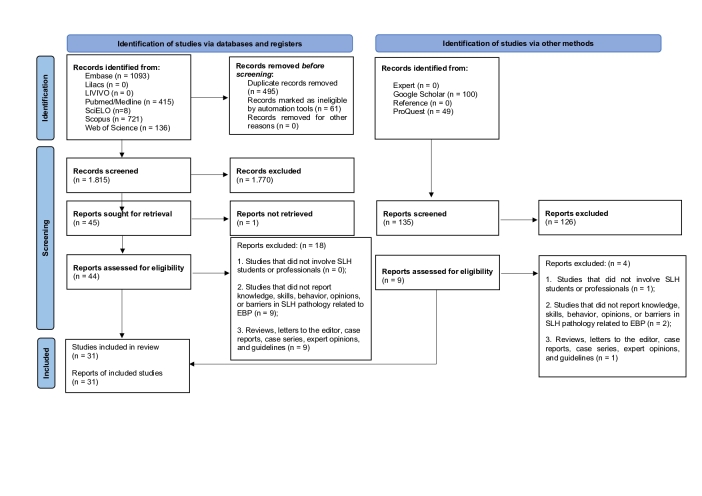
Flowchart of the selection of studies/sources of evidence

The search strategy retrieved 1,950 records from scientific databases and grey literature. The initial screening (Phase 1), based on title and abstract reading, excluded 1,896 records, resulting in 54 studies potentially eligible for full-text reading. One of these could not be retrieved and was therefore not evaluated. Thus, 53 studies had their full texts analyzed (phase 2), of which 22 were excluded for not meeting the previously established eligibility criteria (Appendix 2). At the end of the selection process, 31 studies were included in the qualitative synthesis (Figure 2). No additional studies were identified in the references of the selected works.

*From:* Page et al.^(27)^. For more information, visit: http://www.prisma-statement.org/

### Characteristics of the studies

Table 1 describes the characteristics of the included studies. The participants of the 31 studies analyzed were from different countries, namely: Saudi Arabia, Malaysia, Japan, South Korea, the United States, Canada, Belgium, Australia, New Zealand, the United Kingdom, Ireland, Iran, Brazil, Argentina, Paraguay, Chile, Peru, Colombia, Uruguay, Costa Rica, and the Netherlands. The studies were published between 2004 and 2024. The sample size varied between the studies, ranging from nine^(47)^ to 2,762^(43)^ individuals.

**Table 1:** Characteristics of the studies included in the analysis (n = 31)

**Author, Year, Country**	**Sample characteristics**	**Number of participants (Response rate, %)**	**Instrument used**	**Aspects of Evidence-Based Practice**
Alaidary, Abdulsalam et al.^(2)^, 2020, Saudi Arahia	Speech-language pathologists	n=65* (73,8) *n= 48 participated	Online questionnaire	Favorable attitudes towards EBP, knowledge, behavior, opinions, barriers, and skills
Bennett et al.^(31)^, 2019, Australia	Speech-language pathologists	n= 145 (NR)	Online questionnaire with open-ended responses via Qualtrics	The indispensability of a multidisciplinary team, behaviors, opinions, and barriers
Chan A. K.^(32)^ et al., 2013, Australia	Speech-language pathologists	n= 58 (NR)	Online questionnaire with Likert-scale questions via Qualtrics	Knowledge, skills, behaviors, opinions, and barriers
Cheung et al.^(33)^, 2013, Australia	Speech-language pathologists	n= 772* (13,6) *n= 105 participated	Online questionnaire	Evidence-Based Practice (EBP) requires knowledge, skills, behaviors, opinions, and barriers
Choi et al.^(34)^, 2015, South Korea	Speech-language pathologists	n= 274* (85) *n= 234 participated	Online questionnaire	Generally positive attitude towards PBE, knowledge, skills, behaviors, opinions, and barriers
Chu et al.^(23)^, 2021, Japan and Malaysia	Speech-language pathologists	n= 150* (NR) *n= 145 participated	Online questionnaire with Likert-scale questions	Available resources, demographic variables, cross-cultural comparison, knowledge, skills, behavior, opinions, and barriers
Chu et al.^(35)^, 2022, Malaysia and USA	Speech-language pathology students	n= 69 (NR)	Practical activity and Likert-scale questions	Cross-cultural comparison, relevance of university education, knowledge, skills, behaviors, opinions, and barriers
Cormack, Ailbhe^(36)^, 2010, USA	Speech-language pathologists	n= 85 (NR)	Structured questionnaire	Knowledge, skills, behaviors, opinions, and barriers
Cunningham, B.^(37)^, 2019, Canada	Speech-language pathologists	First survey n=56* (96) *n=54 participated Second survey n=28* (90) *n=25 participated	Online educational module + two questionnaires with Likert-scale questions	Knowledge, skills, behavior, opinions, and barriers
Durieux et al.^(38)^, 2015, Belgium	Speech-language pathologists	n= 2068*; (20) *n= 415 participated	Online questionnaire	Factors influencing the implementation of EBP: knowledge, skills, behavior, opinions, and barriers
Foster, A^(39)^, 2015, Australia	Speech-language pathologists	n= 14 (NR)	Semi-structured interviews	Facilitators of EBP: knowledge, skills, behavior, opinions, and barriers
Fulcher-Rood et al.^(40)^, 2020, USA	Speech-language pathologists	n= 25 (NR)	Semi-structured telephone interviews	Gap between research and practice, knowledge, skill, behavior, opinions and barriers
Gomez et al.^(41)^, 2019, Australia and New Zealand	Speech-language pathologists	n= 138* (79) *n= 109 participated	Online questionnaire with Likert-scale questions	Knowledge, skills, behavior, opinions, and barriers
Greenwell, T. et al.^(21)^, 2021, USA	Speech-language pathologists	n= 324*; (97,8) *n= 317 participated	Online questionnaire	Impact of training on PBE, knowledge, skills, behavior, opinions, and barriers, and the relationship between these barriers
Hegarty, N. et al.^(42)^, 2020, United Kingdom	Speech-language pathologists	n= 21 (NR)	Interviews and focus groups	Factors influencing clinical decision-making include knowledge, skills, behavior, opinions, and barriers
Hoffman, L. M. et al.^(43)^, 2013, USA	Speech-language pathologists	n= 2.762*; (87) *n= 2.394 participated	Online questionnaire	Resources available to support PBE: knowledge, skills, behavior, opinions, and barriers
Mansuri, B. et al.^(5)^, 2020, Iran	Speech-language pathologists	n= 600*; (70) *n=411 participated	Self-administered questionnaire (SLP-EBPQ)	Relationship between educational level and PBE (Problem-Based Learning): Knowledge, skills, behavior, and opinions
McCurtin, A^(44)^, 2015, Ireland	Speech-language pathologists	n= 249 (NR)	Online questionnaire	Knowledge, skills, behavior, opinions, and barriers
Muttiah, N. et al.^(45)^, 2011, USA	Speech-language pathologists	n= 22 (NR)	Qualitative interviews conducted in person and via telephone	Knowledge, skills, behavior, opinions, and barriers
Nail-Chiwetalu et al.^(46)^, 2007, USA	Speech-language pathologists	n= 1.000*; (21) *n= 208 participated	Online questionnaire	Knowledge, skills, behavior, opinions, and barriers
O'Connor, S. et al..^(24)^, 2009, Ireland	Speech-language pathologists	n= 39*; (82,1) *n=32 participated	Mailed questionnaire	Knowledge, skills, behavior, opinions, and barriers
Rojas, C. et al..^(47)^, 2023, Latin America	Speech-language pathologists	n= 22*; (41) *n= 9 participated	Semi-structured interviews conducted via email	Regional realities, knowledge, behavior, opinions, and barriers
Sandham, V. et al..^(48)^, 2021, Australia	Speech-language pathologists	n= 198*; (55,1%) *n= 109 participated	Online questionnaire	Knowledge, skills, behavior, opinions, and barriers
Sandham, V. et al.^(49)^, 2022, Australia	Speech-language pathologists	n= 15 (NR)	Focus groups	Modeling and reflective practice for use in the clinical context: knowledge, skills, behavior, opinions, and barriers
Souza et al.^(1)^, 2024, Brazil	Speech-language pathologists	*n= 122 (NR)	Online questionnaire	Knowledge, skills, behaviors, opinions, and barriers
Spek et al.^(50)^, 2013, Netherlands	Speech-language pathology students	n= 182*; (82%) *n= 149 participated	In-person questionnaire (DMF)	Self-efficacy and task value, knowledge, skills, opinions, and barriers
Thome et al.^(51)^, 2018, USA	Speech-language pathologists	n= 285*; (61,75) *n= 176 participated	Online questionnaire	Knowledge, skills, behaviors, opinions, and barriers
Tohidast et al.^(52)^, 2017, Iran	Speech-language pathologists	n= 200*; (63,5) *n= 127 participated	Online questionnaire	Knowledge, skills, behaviors, opinions, and barriers
Tohidast et al.^(53)^, 2021, Iran	Speech-language pathologists	n= 30*; (46,7) *n= 14 participated	Semi-structured interviews	Opinions and barriers
Vallino-Napoli, L. D. et al.^(54)^, 2004, Australia	Speech-language pathologists	n= 697* (54,2) *n= 378 participated	Mailed questionnaire	Knowledge, skills, behaviors, opinions, and Barriers
Zipoli Jr, R. P. et al.^(25)^, 2005, USA	Speech-language pathologists	n= 488*; (49,2) ^*^n= 240 participated	Mailed questionnaire	Knowledge, skills, behaviors, opinions, and barriers

* n: Number of responses

**Caption:** NR: Not reported; n: Number of invitations sent; SLP-EBPQ: Speech and Language Pathology Evidence-Based Practice Questionnaire; DMF: Dutch Modified Fresno

The participants in the included studies were mostly SLH pathologists, working in a variety of professional settings. Many worked in public and private hospitals^(41)^, including acute stroke units^(39)^, general hospitals^(45)^, hospital facilities, and acute care settings^(24)^. Others were employed in public and private schools^(46)^, encompassing primary, secondary, and higher education schools^(25)^, special schools^(41)^, and school-based settings^(43)^. Professionals were also identified in private clinics and offices^(46)^, independent practice, university clinics, and services provided in clients' homes^(41)^. There were also participants working in community settings, such as community health centers^(41)^, and in geriatric and residential care settings^(31)^, such as nursing homes^(46)^. Some worked in government organizations and public services^(33)^, including programs such as the Infant Hearing Program in Canada^(37)^ and health and social care structures such as the HSCTs in Northern Ireland^(42)^. Studies also mentioned non-governmental organizations (NGOs)^(41)^ and voluntary agencies focused on serving people with intellectual disabilities and autism spectrum disorder (ASD)^(24)^ and rehabilitation centers^(46)^. Finally, some professionals worked in other contexts, such as home health services, early intervention programs, as independent subcontractors^(46)^, or simply referred to as the "unit" or "department" where they worked^(53)^.

Several studies included in this review based the development of their data collection instruments on previous research, especially in the context of EBP in SLH pathology. Despite methodological variations, the questionnaires share recurring themes, such as attitudes, skills, sources used, and perceived barriers to the implementation of EBP.

Many of the questionnaires were adapted, such as those developed by Zipoli and Kennedy, Jette et al., Nail-Chiwetalu et al., Vallino-Napoli and Reilly and Funk et al., frequently used in studies with SLH professionals and related areas. These instruments served as a basis for research in different contexts, such as in the studies by Alhaidary et al., Chu et al., and Greenwell et al., who adapted specific items according to the local reality and investigative objectives.

Some studies opted to develop their own instruments based on previous models, such as Cheung et al., who used an online questionnaire adapted from SLH pathology for the context of autism, and Souza et al., who adapted instruments from physiotherapy for the Brazilian reality. Even in qualitative studies, such as that of Sandham et al., the interviews were guided by scripts based on previous quantitative data, demonstrating integration between methodological approaches.

### Risk of bias in studies

**Figure 3:**
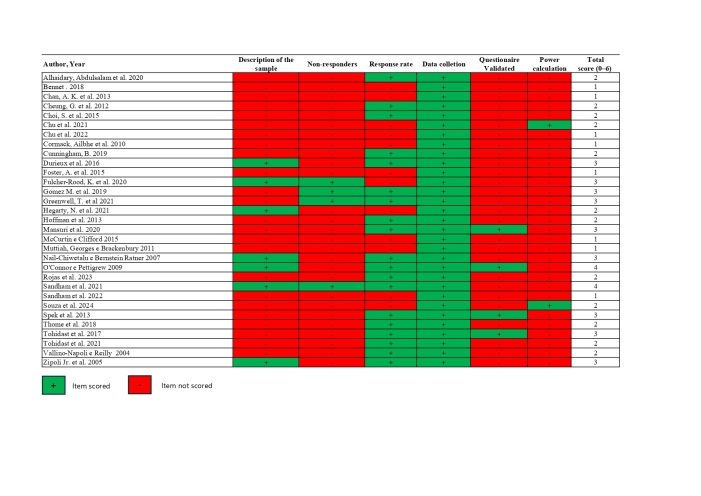
Risk of bias assessment of the included studies

The scores assigned to the included studies ranged from 1 to 4 points, on a scale of 0 to 6, according to the criteria used to assess the risk of bias. Most studies scored between 1 and 3 points, while two studies achieved 4 points^(24,49)^. The most frequently met criteria were data collection focusing on EBP aspects such as attitudes, skills, opinions, barriers, and behaviors, followed by sample description and response rate. On the other hand, the least frequently reported criteria included the absence of information on non-respondents, the lack of validation of the instruments used, and the absence of a description of the sample size calculation. Figure 3 presents the individual scores of the studies according to the evaluated criteria.

### Individual study results

#### EBP knowledge

The studies analyzed reveal that SLH pathologists and students often have a partial understanding of EBP, frequently reduced to the exclusive use of scientific research results, with little appreciation for clinical experience and patient preferences^(22,40,47)^. Only a minority recognizes the three pillars that make up EBP. In different surveys, only 8%, 14%, and 28% of participants correctly identified the three components^(40,51,54)^. Furthermore, some professionals mistakenly believe that EBP requires the use of the “latest published evidence,” and not necessarily the best available evidence^(47)^.

Professionals with postgraduate training tend to perform better on EBP tests and have greater familiarity with its central concepts^(5,23)^. However, gaps are evident in fundamental technical skills even among them, such as statistical interpretation, critical evaluation of the literature, and the search for relevant studies. In Brazil, only 31.1% of participants stated that they understood statistical analyses, and most reported not having received formal training for the critical evaluation of scientific studies^(1)^. These difficulties are exacerbated by the language barrier, since most relevant publications are available only in English, a language that is still an obstacle for many professionals^(1,20)^.

**Figure 4:**
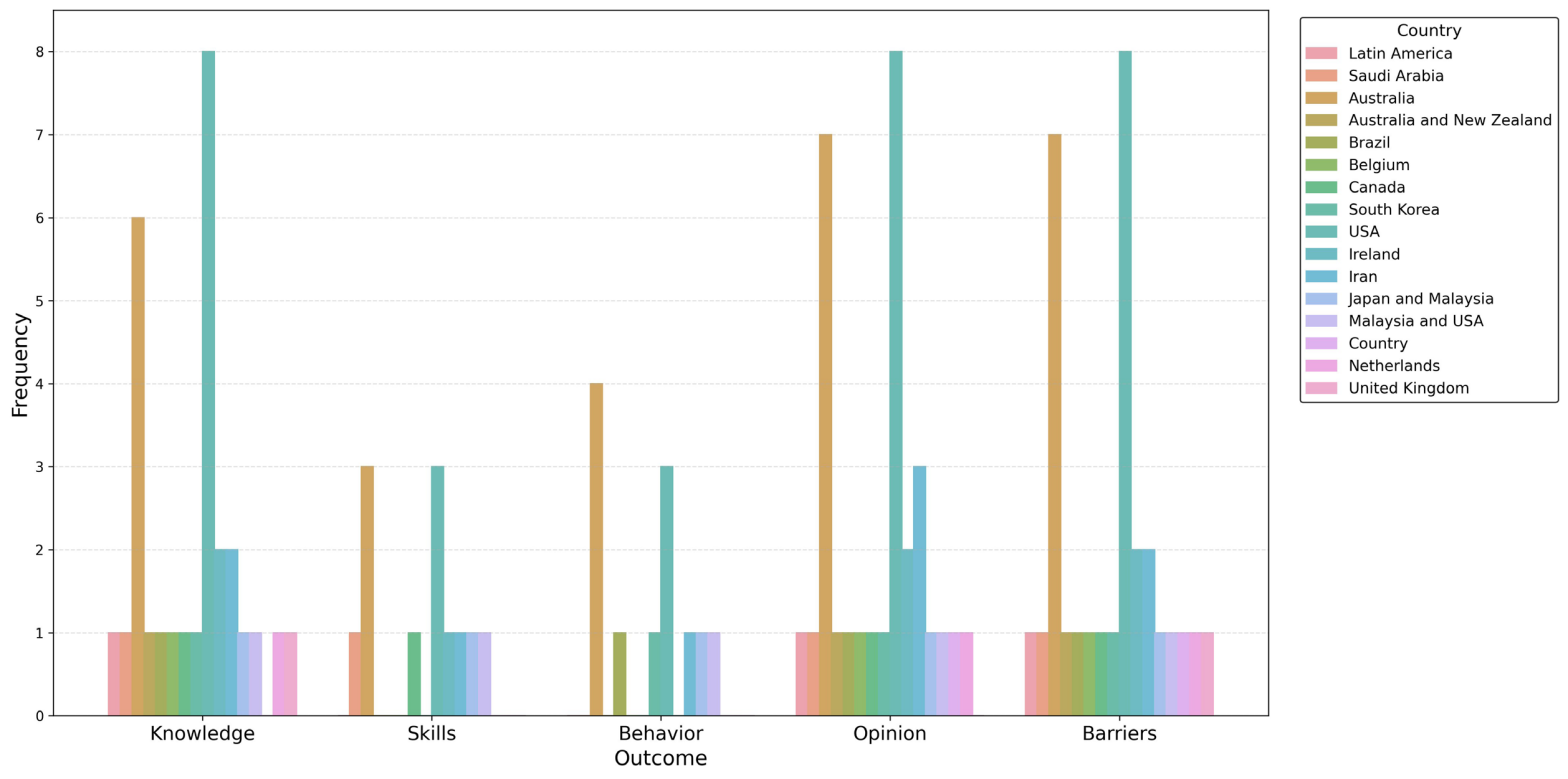
Frequency of outcomes by country

Figure 4 illustrates the outcomes most frequently reported in relation to countries, and Appendix 3 presents all aspects included in each study. Regarding the distribution of the aspects of EBP investigated in the studies, the most frequently analyzed domains were knowledge, opinions, and barriers, each reported in studies from 14 countries. The skills domain was addressed in 10 countries, while behavior was the least investigated, present in studies from nine countries. It is noteworthy that Brazil, the United States, and Australia concentrated the greatest variety of domains evaluated, while countries such as Ireland and Iran focused mainly on knowledge and barriers. These findings highlight the conceptual and methodological heterogeneity among international studies on EBP and reinforce the importance of broad mappings, such as the present one, to understand the diversity of approaches, contexts, and levels of implementation of this practice among SLH pathologists.

#### EBP skills and behaviors

Most studies have shown that, even with prior training, professionals feel the need to improve their skills in EBP. In the United States, about 70% reported this need, while in Brazil, this number was even more significant (97.1%)^(1,2,22,50)^. Recurring technical deficiencies include difficulties in the methodological analysis of studies, in the interpretation of statistical data, and in the use of efficient search strategies in scientific databases^(35)^. Such difficulties are more prevalent among professionals at the beginning of their careers: in Ireland, for example, 72.8% of beginning therapists reported difficulties with statistics, compared to 42.9% of more experienced professionals^(24)^.

Another relevant point refers to the sources of information used in clinical practice. Although the importance of scientific research is recognized, most professionals still rely predominantly on their own clinical experience (reported by up to 99.6% of respondents) and on the opinions of colleagues (up to 78.7%)^(5,22,25,48)^. The use of high-level scientific databases, such as Cochrane or SpeechBite, is still limited, especially in Brazil, where platforms such as SciELO (33.6%) and Google Scholar (23.2%) are more frequently used, reflecting limited access, linguistic barriers, and unfamiliarity with the tools^(1,52)^.

There is evidence that academic training positively influences the adoption of EBP. Exposure to research during undergraduate and postgraduate studies and clinical fellowship year is associated with greater appreciation and use of EBP in practice^(2,22,25,34)^. However, studies indicate that this exposure tends to decrease after the initial training period^(25)^. EBP use has been more incorporated into therapeutic decision-making than in the evaluation phases, being frequently used in clinical uncertainty or to validate previously adopted conduct^(40)^.

#### Opinions on EBP

Studies generally show that SLH pathologists maintain positive attitudes toward EBP^(36,52)^. Professionals from countries such as Malaysia, Iran, Australia, and Brazil recognize the benefits of the practice for the quality of care and the effectiveness of clinical decisions^(1,5,23,33)^. In Brazil, 89.3% of respondents stated that EBP improves patient care, and 86.8% consider it useful for treatment^(1)^. Prior exposure to EBP during training fosters more favorable attitudes^(2,22)^.

Despite the receptiveness, there is still conceptual confusion, mainly regarding the integration between scientific evidence, clinical experience, and patient values. Many professionals continue to associate EBP exclusively with research, disregarding the other components^(39,47)^. The perception of overload associated with the use of EBP was also mentioned, especially by those with less English proficiency or who work in environments without institutional support^(1)^.

Institutional support is a critical factor. Although some report valuing EBP in the workplace, the perception that it effectively guides clinical decisions is still limited. In Brazil, almost half of the professionals pointed to the lack of support among colleagues as a barrier, and many reported insecurity in recommending the use of evidence, especially in front of hierarchical superiors^(1,33)^.

#### EBP barriers

Barriers to the application of EBP are consistent across studies and range from individual limitations to organizational factors. The most frequent is the lack of time, mentioned by 91% of school SLH pathologists in the US^(43)^, 71.9% in Ireland^(24)^, 62% in Iran^(53)^, and 27.9% in Brazil, especially among professionals with less training time^(1)^.

Technical difficulties, such as understanding statistics, evaluating the quality of studies, and applying them to individual cases, were widely reported. Up to 78% of professionals in the UK and Ireland reported problems with statistics, and in the US, this rate reached 33%^(22,24)^. In Brazil, more than half of those interviewed indicated difficulty in applying research results to clinical practice^(1)^.

Limited access to scientific literature and low familiarity with specialized databases also hinder the implementation of EBP. Many professionals reported difficulties in locating relevant articles and low use of platforms such as Cochrane, PubMed, and SpeechBite^(1,22,33)^. The language barrier is an additional challenge, particularly affecting professionals who do not master English^(1,47)^.

The absence of formal institutional guidelines, the lack of encouragement for EBP, and the lack of support among colleagues stand out among organizational barriers. Only 11% of professionals in the US reported working in institutions with specific EBP guidelines^(43)^. In Brazil, 48.4% cited the absence of collective support as an obstacle^(1)^.

Finally, limitations related to scientific production were mentioned, such as low replicability, methodological weakness, and reduced practical applicability. These criticisms were identified in studies conducted in Ireland, Australia, Brazil, and South Korea^(1,2,24,32,38-44,49,53)^, reinforcing the importance of producing more solid, relevant, and contextualized evidence for clinical SLH practice.

### Reporting bias

The funnel plot analysis of publication bias was not performed, as this approach requires a meta-analysis, which was not applicable in this study. To minimize the possibility of publication bias, a comprehensive search strategy was adopted, including seven databases, grey literature, and a database in a language other than English (LILACS).

## DISCUSSION

Evidence-based SLH therapy has been increasingly discussed and disseminated in recent years. The study results, where most participants conceptualize EBP primarily as the use of scientific publications and research^(47)^, are aligned with a trend observed in several studies. Many SLH pathologists and other health professionals limit EBP to research evidence, neglecting the integration of the professional's clinical experience and the client's preferences and values^(40,45,47)^. This restricted perception contrasts with widely accepted definitions, which consider EBP as the conscious, explicit, and judicious integration of the best available external research evidence, the best internal evidence from clinical practice, and the preferences of fully informed patients. The American Speech-Language-Hearing Association (ASHA), for example, emphasizes this triad in its guidelines^(6)^.

Exposure to EBP during undergraduate studies and, especially, in the first years of clinical practice, such as the clinical improvement period, has proven to be a relevant factor for the incorporation of EBP into professional routine^(1,2,22,25,34)^. Professionals with greater training or training directed at EBP tend to report more confidence in the critical evaluation of evidence and its use in clinical decision-making^(2,34,53)^. Those with less English proficiency were less familiar with EBP principles, demonstrating that the language barrier is still an important obstacle to accessing the most impactful international scientific literature^(1)^.

Despite generally positive attitudes towards EBP, obstacles persist that hinder its implementation. Lack of time continues to be one of the most cited barriers, attributed to high workloads and pressure for productivity^(1,2,24,38-44,49,53^ . The perception of insufficient time was more pronounced among professionals with less experience, suggesting that experience can mitigate this difficulty. However, the literature is not unanimous regarding this association, with some studies indicating that this perception is independent of the length of experience^(24)^.

Other recurring obstacles include the scarcity of robust studies directly applicable to clinical practice, difficulty in interpreting and applying research findings^(1,2,22,24,33,38,40,44,49,53)^, and the perception that articles lack clarity and practical applicability. Many professionals report that studies do not engage with the realities of their work contexts or with the specific needs of patients^(49)^.

Moreover, the lack of institutional support and an organizational culture geared towards valuing scientific evidence weakens adherence to EBP. In several contexts, EBP is perceived as a bureaucratic requirement, without effective integration with clinical routines. There is also limited access to databases, specialized search tools, and up-to-date resources, as well as the cost of obtaining scientific materials, further hindering EBP, especially in low- and middle-income countries^(46)^.

The language barrier further limits access to the most internationally relevant literature. In this scenario, academic training plays an important role: SLH pathologists with postgraduate degrees report fewer difficulties in adopting EBP, reinforcing that familiarity with scientific research can mitigate some of these barriers^(1,38,47)^.

The risk of bias analysis revealed methodological weaknesses in the included studies. Most obtained low scores (1 to 3 on a scale of 0 to 6), which points to important limitations in the quality of the evidence. Only two studies reached 4 points^(24,49)^, and none reached the maximum score. Although aspects such as sample description, response rate, and focus on EBP components were frequently addressed, fundamental elements of methodological rigor were lacking. The absence of information on non-respondents, the lack of instrument validation, and the omission of sample size calculations were recurring issues, compromising the reliability and generalizability of the findings. These methodological flaws limit the reliability of the available evidence and reinforce the need for more rigorous future studies, with special attention to the design, instrument validity, and transparent data presentation.

The quality of the included studies was significantly heterogeneous. Although some have good methodological foundations, many lack scientific rigor due to either the absence of controlled designs^(24)^ or insufficient details that make the replication of interventions unfeasible. Moreover, the literature in the area often does not provide sufficient practical information (such as materials used, intervention protocols, and clinical adaptation criteria), making the real-world implementation of the evidence difficult^(40,49)^. This scenario contributes to the skepticism of many clinicians regarding the applicability of academic research in their realities.

The gap between research and practice remains one of the main challenges of EBP. This disconnect is fueled by academic productions poorly aligned with the needs of practice and by professionals who feel unprepared or unsupported to integrate scientific evidence into their work^(39,40,44,47)^. Strategies identified as promising include producing studies that are more applicable to real clinical practice^(1,40,47)^, detailed descriptions of interventions^(40,49)^, collaboration between researchers and clinicians in research planning, and the creation of easily accessible and quickly consulted resources, such as clinical abstracts and updated guidelines^(37,40,42,45)^.

It is also essential to promote continuous training in EBP, focusing on search skills, critical appraisal, and practical application, and foster work environments that value and support the use of evidence, through discussion groups and mentoring^(1,40,44)^. It is important to emphasize that EBP is not limited to published research: it also involves clinical expertise and patient preferences. Integrating these three points is fundamental for EBP to be truly effective and sustainable.

This study has limitations that should be considered. The sample, obtained through online questionnaires and social networks, may have been subject to self-selection bias, favoring the participation of professionals more familiar with technology or more interested in the topic. The use of self-reporting may have introduced social desirability bias, leading participants to respond more aligned with what is socially expected, in addition to the possibility of memory errors or subjective judgment.

Open-ended and semi-structured questions, while useful for exploring perceptions, may not have captured all dimensions of EBP or explored in depth the reasons why certain barriers were mentioned. Sample homogeneity in terms of demographic profile and training also limits the generalization of the findings to the entire population of SLH pathologists.

Finally, the study focused on the perceptions and attitudes of the participants, not directly assessing the quality of clinical decisions or distinguishing between effective use of EBP and the perception of its use. Future investigations should consider mixed methodological approaches, combining questionnaires with observations in clinical settings and analysis of therapeutic decisions to deepen the understanding of the actual use of EBP in professional practice.

Given this scenario, the findings of this research reinforce that EBP appreciation, if not accompanied by consistent training and structural policies, is ineffective. Coordinated action between universities, health institutions, and professional councils is essential so that training in EBP is continuously and effectively integrated into the professional trajectory. In parallel, it is urgent to raise the methodological rigor of research produced in the area, bringing it closer to the demands of real clinical practice.

## CONCLUSION

It can be concluded, therefore, that although SLH pathologists generally demonstrate positive attitudes toward EBP, significant gaps persist regarding knowledge, skills, and practical application. Many professionals still perceive EBP in a limited way, valuing exclusively scientific evidence without adequately integrating clinical experience and patient preferences, which are central elements to the concept of EBP.

The main barriers identified were lack of time, low familiarity with search methods and critical appraisal, insufficient English proficiency, and lack of institutional support. These points are repeated in different contexts and directly impact the efficient implementation of EBP. Furthermore, the included studies had heterogeneous methods, with low scores on the risk of bias and a scarcity of validation of the instruments used, which reduces overall confidence in the available evidence.

Despite this, prior exposure to EBP during academic training proved to be an important and promising factor for better attitudes and behaviors related to practice, suggesting that investment in specific training can mitigate some of the barriers faced and bring more confidence to interested professionals.

## Appendix 1 Search strategies in databases and grey literature

**Table:**

**Database**	**Search** (November 19th, 2024)
**Embase**	#1. 'evidence based'/exp OR 'evidence based' OR 'evidence-based practice'/exp OR 'evidence-based practice' OR 'evidence based practice'/exp OR 'evidence based practice' OR 'evidence based medical practice'/exp OR 'evidence based medical practice' OR 'evidence-based medicine'/exp OR 'evidence-based medicine' OR 'evidence based medicine'/exp OR 'evidence based medicine'
	#2. 'attitude' OR 'attitudes' OR 'sentiment' OR 'sentiments' OR 'opinions' OR 'opinion' OR 'behavior' OR 'self-efficacy' OR 'knowledge' OR 'perception' OR 'research' OR 'barrier' OR 'skill' OR 'intention' OR 'belief' OR 'self-reported practice' OR 'education' OR 'interest' OR 'resources' OR 'participation' OR 'abilit' OR 'implementation' OR 'implementing' OR 'access' OR 'problem'
	#3. 'speech therapy'/exp OR 'logopedic education' OR 'logopedic training' OR 'speech education' OR 'speech training' OR 'therapy, speech' OR 'speech therapy' OR 'speech, language and hearing sciences' OR 'speech-language pathology' OR 'speech language pathology' OR 'language pathology' OR 'speech pathology'
	#4. #1 AND #2 AND #3
	#5. #4 AND [embase]/lim NOT ([embase]/lim AND [medline]/lim)
**LILACS**	#1.MH:"Prática Clínica Baseada em Evidências" OR "Prática Clínica Baseada em Evidências" OR "Assistência Sanitária Baseada em Evidência" OR "Assistência Sanitária Baseada em Evidências" OR "Atenção à Saúde Baseada em Evidências" OR "Atenção à Saúde Baseada na Evidência" OR "Cuidado à Saúde Baseado em Evidências" OR "Cuidados de Saúde Baseados em Evidências" OR "Cuidados de Saúde Baseados na Evidência" OR "Prática Médica Baseada em Evidências" OR "Saúde Pública Baseada em Evidência" OR "Saúde Pública Baseada em Evidências" OR "Saúde Pública Baseada na Evidência" OR "Evidence-Based Practice" OR "Práctica Clínica Basada en la Evidencia" OR MH:H02.249$
	#2. MH:Atitude OR Atitude OR Atitudes OR Opinião OR Opiniões OR "Sentimento" OR "Actitud" OR "Opiniones" OR "Sentimiento" OR "Attitude" OR "Attitudes" OR "Opinion" OR "Opinions" OR "Sentiment" OR "Sentiment" OR "Sentiments" OR "behavior" OR "self-efficacy" OR "self-efficacy" OR "knowledge" OR "perception" OR "research" OR "barrier" OR "skill" OR "intention" OR "belief" OR "self-reported practice" OR "education" OR "interest" OR "resources" OR OR "participation" OR "abilit" OR "implementation" OR "implementing" OR "access" OR "problem" OR MH:F01.100$
	#3.MH:Fonoaudiologia OR Fonoaudiologia OR "Speech, Language and Hearing Sciences" OR Fonoaudiología OR "Ciência da Fala e Audição" OR "Ciência da Fonação e Audição" OR "Estudo da Fala e Audição" OR "Estudo da Fala e da Audição" OR "Estudo da Fonação e Audição" OR "Estudos da Fala e Audição" OR "Estudos da Fala e da Audição" OR "Estudos da Fonação e Audição" OR "Estudos da Fonação e da Audição" OR "Patologia da Fala e Linguagem e Audiologia" OR MH:SH1.020.020.040.045$ OR MH:"Patologia da Fala e Linguagem" OR "Speech-Language Pathology" OR "Patología del Habla y Lenguaje" OR Patologia da Fala" OR "Patologia da Linguagem" OR MH:H02.010.750$
	#4. #1 AND #2 AND #3
**LIVIVO**	("Evidence-Based Practice" OR "Evidence Based Practice" OR "Evidence-Based Health Care" OR "Evidence Based Health Care" OR "Evidence-Based Healthcare" OR "Evidence Based Healthcare" OR "Evidence Based Health Care Management" OR "Evidence Based Healthcare Management" OR "Evidence-Based Medicine" OR "Evidence Based Medicine") AND ("Attitude" OR "Attitudes" OR "Sentiment" OR "Sentiments" OR "Opinions" OR "Opinion" OR "behavior" OR "self-efficacy" OR "self-efficacy" OR "knowledge" OR "perception" OR "research" OR "barrier" OR "skill" OR "intention" OR "belief" OR "self-reported practice" OR "education" OR "interest" OR "resources" OR "participation" OR "abilit" OR "implementation" OR "implementing" OR "access" OR "problem") AND ("Speech Therapy" OR "Speech Therapies" OR "Speech, Language and Hearing Sciences" OR "Speech-Language Pathology" OR "Speech Language Pathology" OR "Language Pathology" OR "Speech Pathology")
**PubMed/ Medline**	#1. "Evidence-Based Practice"[Mesh] OR "Evidence Based Practice" OR "Evidence-Based Health Care" OR "Evidence Based Health Care" OR "Evidence-Based Healthcare" OR "Evidence Based Healthcare" OR "Evidence Based Health Care Management" OR "Evidence Based Healthcare Management" OR "Evidence-Based Medicine"[Mesh] OR "Evidence Based Medicine"
	#2. "Attitude"[Mesh] OR "Attitudes" OR "Sentiment" OR "Sentiments" OR "Opinions" OR "Opinion" OR "behavior" OR "self-efficacy" OR "self-efficacy" OR "knowledge" OR "perception" OR "research" OR "barrier" OR "skill" OR "intention" OR "belief" OR "self-reported practice" OR "education" OR "interest" OR "resources" OR "participation" OR "abilit" OR "implementation" OR "implementing" OR "access" OR "problem"
	#3. "Speech Therapy"[Mesh] OR "Speech Therapies" OR "Speech, Language and Hearing Sciences" OR "Speech-Language Pathology"[Mesh] OR "Speech Language Pathology" OR "Language Pathology" OR "Speech Pathology"
	#4. #1 AND #2 AND #3
**SciELO**	"Evidence-Based Practice" OR "Evidence Based Practice" OR "Evidence-Based Health Care" OR "Evidence Based Health Care" OR "Evidence-Based Healthcare" OR "Evidence Based Healthcare" OR "Evidence Based Health Care Management" OR "Evidence Based Healthcare Management" OR "Evidence-Based Medicine" OR "Evidence Based Medicine" (Topic) AND "Attitude" OR "Attitudes" OR "Sentiment" OR "Sentiments" OR "Opinions" OR "Opinion" OR "behavior" OR "self-efficacy" OR "self-efficacy" OR "knowledge" OR "perception" OR "research" OR "barrier" OR "skill" OR "intention" OR "belief" OR "self-reported practice" OR "education" OR "interest" OR "resources" OR "participation" OR "abilit" OR "implementation" OR "implementing" OR "access" OR "problem" (Topic) AND "Speech Therapy" OR "Speech Therapies" OR "Speech, Language and Hearing Sciences" OR "Speech-Language Pathology" OR "Speech Language Pathology" OR "Language Pathology" OR "Speech Pathology" (Topic)
**Scopus**	(TITLE-ABS-KEY (( "Evidence-Based Practice" OR "Evidence Based Practice" OR "Evidence-Based Health Care" OR "Evidence Based Health Care" OR "Evidence-Based Healthcare" OR "Evidence Based Healthcare" OR "Evidence Based Health Care Management" OR "Evidence Based Healthcare Management" OR "Evidence-Based Dentistry" OR "Evidence Based Dentistry" OR "Evidence-Based Medicine" OR "Evidence Based Medicine" )) AND TITLE-ABS-KEY (( "Attitude" OR "Attitudes" OR "Sentiment" OR "Sentiments" OR "Opinions" OR "Opinion" OR "behavior" OR "self-efficacy" OR "self-efficacy" OR "knowledge" OR "perception" OR "research" OR "barrier" OR "skill" OR "intention" OR "belief" OR "self-reported practice" OR "education" OR "interest" OR "resources" OR "participation" OR "abilit" OR "implementation" OR "implementing" OR "access" OR "problem" )) AND TITLE-ABS-KEY (( "Dentists" OR "dentist" )))
**Web of Science**	"Evidence-Based Practice" OR "Evidence Based Practice" OR "Evidence-Based Health Care" OR "Evidence Based Health Care" OR "Evidence-Based Healthcare" OR "Evidence Based Healthcare" OR "Evidence Based Health Care Management" OR "Evidence Based Healthcare Management" OR "Evidence-Based Medicine" OR "Evidence Based Medicine" (Topic) and "Attitude" OR "Attitudes" OR "Sentiment" OR "Sentiments" OR "Opinions" OR "Opinion" OR "behavior" OR "self-efficacy" OR "self-efficacy" OR "knowledge" OR "perception" OR "research" OR "barrier" OR "skill" OR "intention" OR "belief" OR "self-reported practice" OR "education" OR "interest" OR "resources" OR "participation" OR "ability" OR "implementation" OR "implementing" OR "access" OR "problem" (Topic) and "Speech Therapy" OR "Speech Therapies" OR "Speech, Language and Hearing Sciences" OR "Speech-Language Pathology" OR "Speech Language Pathology" OR "Language Pathology" OR "Speech Pathology" (Topic) |
**Google Scholar**	"Evidence-Based Practice" AND "Attitude" OR "Sentiment" OR "Opinions" OR "behavior" AND "Speech Therapy" OR "Speech-Language Pathology"
**ProQuest**	"Evidence-Based Practice" OR "Evidence Based Practice" OR "Evidence-Based Health Care" OR "Evidence Based Health Care" OR "Evidence-Based Healthcare" OR "Evidence Based Healthcare" OR "Evidence Based Health Care Management" OR "Evidence Based Healthcare Management" OR "Evidence-Based Medicine" OR "Evidence Based Medicine" (Topic) AND "Attitude" OR "Attitudes" OR "Sentiment" OR "Sentiments" OR "Opinions" OR "Opinion" OR "behavior" OR "self-efficacy" OR "self-efficacy" OR "knowledge" OR "perception" OR "research" OR "barrier" OR "skill" OR "intention" OR "belief" OR "self-reported practice" OR "education" OR "interest" OR "resources" OR "participation" OR "abilit" OR "implementation" OR "implementing" OR "access" OR "problem" (Topic) AND "Speech Therapy" OR "Speech Therapies" OR "Speech, Language and Hearing Sciences" OR "Speech-Language Pathology" OR "Speech Language Pathology" OR "Language Pathology" OR "Speech Pathology" (Topic)

## Appendix 2 Excluded articles and reason for exclusion (n = 22)

**Table:**

**Author, Year**	**Reason for exclusion**
Abrams et al., 2005^1^	3
Alary Gauvreau et al., 2019^2^	3
Allison, 2021^3^	3
Baker et al., 2011^4^	3
Beushausen, 2005^5^	3
Beushausen, 2014^6^	3
Boisvert et al., 2017^7^	3
Borgelt et al., 2015^8^	3
Borgetto et al., 2016^9^	2
Brackenbury et al., 2008^10^	3
Brock, 2025^11^	2
Campbell et al., 2017^12^	2
Georgieva et al., 2011^13^	3
Gomez et al., 2011^14^	2
Guo et al., 2008^15^	2
Harding et al., 2014^16^	1
Kwok et al., 2022^17^	2
Lof, 2011^18^	2
Meline, 2003^19^	2
Mullen, 2005^20^	2
Ratner, 2005^21^	2
Trembath et al, 2016^22^	2

**Caption:** 1. Studies that did not involve SLH students or professional; 2. Studies that did not report knowledge, skills, behavior, opinions, or barriers in SLH pathology related to EBP; 3. Reviews, letters to the editor, case reports, case series, expert opinions, and guidelines.

**REFERENCES FOR** **APPENDIX 2**

1. Abrams HB, McArdle R, Chisolm TH. From outcomes to evidence: establishing best practices for audiologists. Semin Hear. 2005;26(4):207–13.

2. Alary Gauvreau C, le Dorze G, Kairy D, Croteau C. Evaluation of a community of practice for speech-language pathologists in aphasia rehabilitation: a logic analysis. BMC Health Serv Res. 2019;19(1):530.

3. Allison LHZ. Evidence-Based Practice for Speech-Language Pathologists: A Survey of Access and Implementation [dissertation]. Auburn: Auburn University; 2021.

4. Baker E, McLeod S. Evidence-based practice for children with speech sound disorders: Part 2. Application to clinical practice. Lang Speech Hear Serv Sch. 2011;42(2):140–51.

5. Beushausen U. “Evidence-based practice” in speech pathology – myth and reality. Forum Logopädie. 2005;19(2):6-11.

6. Beushausen U. Evidence-based speech-language pathology: prospects and risks. L.O.G.O.S. Interdisziplinär. 2014;22(2):96-104.

7. Boisvert I, Clemesha J, Lundmark E, Crome E, Barr C, McMahon CM. Decision-making in audiology: balancing evidence-based practice and patient-centered care. Trends Hear. 2017;21:1–14.

8. Borgelt T. When there is no evidence available: evidence-based practice scenarios in day-to-day speech therapy care. Forum Logopädie. 2015;29(1):24–29.

9. Borgetto B, Spitzer L, Pfingsten A. The research pyramid: how to make evidence useful for the practice of speech and language therapy. Forum Logopädie. 2016;30(1):24-28.

10. Brackenbury T, Burroughs E, Hewitt LE. A qualitative examination of current guidelines for evidence-based practice in child language intervention. Lang Speech Hear Serv Sch. 2008;39(1):96–109.

11. Brock AS. Using case-based learning to teach evidence-based practice: a pilot study. Clin Teach. 2025;22(1):e13842.

12. Campbell WN, Douglas NF. Supporting evidence-based practice in speech-language pathology: a review of implementation strategies for promoting health professional behavior change. Evid Based Commun Assess Interv. 2017;11(3–4):43–51.

13. Georgieva D, Stefanovska A. Evidence-based assessment of voice disorders: a theoretical overview and model. J Spec Educ Rehabil. 2011;12(1–2):32-39.

14. Gomez M, McCabe P, Purcell A. A survey of the clinical management of childhood apraxia of speech in the United States and Canada. J Commun Disord. 2022;96:106181.

15. Guo R, Bain BA, Willer J. Results of an assessment of information needs among speech-language pathologists and audiologists in Idaho. J Med Libr Assoc. 2008;96(2):138–144.

16. Harding KE, Porter J, Horne-Thompson A, Donley E, Taylor NF. Not enough time or a low priority? Barriers to evidence-based practice for allied health clinicians. J Contin Educ Health Prof. 2014;34(4):224–31.

17. Kwok EY, Moodie ST, Cunningham BJ, Oram Cardy J. Barriers and facilitators to implementation of a preschool outcome measure: an interview study with speech-language pathologists. J Commun Disord. 2022;95:106160.

18. Lof GL. Science-based practice and the speech-language pathologist. Int J Speech Lang Pathol. 2011;13(3):189–96.

19. Meline T, Paradiso T. Evidence-based practice in schools. Lang Speech Hear Serv Sch. 2003;34(4):273–83.

20. Mullen R. Survey tests members’ understanding of evidence-based practice: a systematic approach to earmold selection. Leader. 2005;10(15):4-14.

21. Ratner NB. Evidence-based practice in stuttering: some questions to consider. J Fluency Disord. 2005;30(3):163–88.

22. Trembath D, Hawtree R, Arciuli J, Caithness T. What do speech-language pathologists think parents expect when treating their children with autism spectrum disorder? Int J Speech Lang Pathol. 2016;18(3):250-59.

## Appendix 3 Description of characteristics related to knowledge, skills, attitudes, opinions, and barriers reported by each study (n = 31)

**Table:**

**Name, Year, Country**	**Knowledge and skills**	**Behaviors**	**Opinions**	**Barriers**
Alaidary, Abdulsalam et al.^(2)^, 2020, Saudi Arabia	93.8% completed a research methodology course;	The main sources of information for clinical decision-making were personal clinical experience, clinical practice guidelines, Internet resources, textbooks, and research studies.	90% agreed that it is important to allocate time for Evidence-Based Practice (EBP) and that its implementation should be encouraged;	60% reported not having time to read research literature at work;
	87.5% were exposed to scientific articles;	Specific training in Evidence-Based Practice (EBP) increased the use of research studies to guide clinical decision-making.	60.4% consider research findings relevant to daily practice;	52.1% had access to online research tools at work;
	79.2% graduated with an understanding and knowledge of Evidence-Based Practice (EBP);		41.7% believe that EBP will increase their workload.	The application of research findings in practice was perceived as challenging, with 37.5% agreeing and 33.3% being neutral.
	66.7% received training in EBP;			
	66.7% feel comfortable reading scientific articles.			
Bennett et al.^(31)^, 2019, Australia	They recognize the importance of standardized tools and objective assessments;	Due to time constraints, there is a high use of screening tools rather than comprehensive assessments;	They consider that the “best care” (research-based) should be balanced with “responsible care,” especially for frail older adults with cognitive impairments;	Access to, and the dynamics of, an effective multidisciplinary team and a professional support network;
	The clinical usefulness of tools developed for adults is questioned, as older adults have comorbidities and complex needs.	Educating staff, family members, and caregivers is essential in Evidence-Based Practice, especially in community and residential care settings with limited management resources.	The study proposes an expanded model of Evidence-Based Practice decision-making for older adults, considering the clinical context and the role of the care team.	Lack of time and limited resources;
				The lack of validated assessment and therapy tools for complex older adult populations is a barrier;
			
				Limited resources, geographic location, and low prioritization restrict continuing education in Speech-Language Pathology for older adults, further aggravated by costs and accessibility issues.
Chan A. K. et al.^(32)^, 2013, Australia	Most report having the fundamental skills to search for and critically appraise the literature;	In the absence of high-quality evidence, 98% rely on their own clinical experience;	96% of speech-language pathologists sometimes or always used the therapy approach they believed to be the most effective;	88% believed there was a lack of high-quality evidence;
	Lack of training and limited access to specialized workshops hinder the provision of “ideal” treatment.	Other sources used when high-quality evidence is lacking include consulting textbooks (91%), colleagues (89%), and participating in professional development events (83%).	Management choices are influenced by the clinician’s clinical experience, patient-centered reasons, and the presence of external evidence.	86% reported that evidence is not always accessible;
				81% reported a lack of time to read the literature;
				Lack of funding for formal training programs was also mentioned.
Cheung et al.^(33)^, 2013, Australia	97% agreed that the application of Evidence-Based Practice is necessary;	23% believe that most speech-language pathologists apply Evidence-Based Practice in the care of children with Autism Spectrum Disorder;	Evidence-Based Practice is viewed as fundamental for effective and ethical practice;	Lack of understanding from management;
	64% received training in Evidence-Based Practice;	10% rarely access Evidence-Based Practice resources, while 46% access them weekly;	97% agree that Evidence-Based Practice is necessary in Speech-Language Pathology practice;	44% do not feel they have time to review the literature before making clinical decisions;
	48% had adequate training to use electronic research search tools efficiently;	Professionals rely on clinical judgment and experience due to the lack of high-quality research on autism.	41% perceive a gap between research and practice;	18% reported that managing the waiting list is prioritized over evidence-based interventions;
	76% regularly search for research evidence;		82% indicated that Evidence-Based Practice is supported in their workplaces;	28% indicated that they lack the necessary electronic tools to find appropriate research;
	Speech-language pathologists with more than 10 years of experience feel more confident recommending the use of Evidence-Based Practice to colleagues, especially junior professionals.		66% indicated that service decisions are based on research evidence;	34% indicated that their workplaces do not have sufficient funding for Evidence-Based Practice activities.
			A common criticism is that managers and administrators do not understand the importance of Evidence-Based Practice.	
Choi et al.^(34)^, 2015, North Korea	A positive attitude toward Evidence-Based Practice is associated with exposure to research and practice during undergraduate education and internships;	Clinical experience guides decisions more than research studies;	Generally positive attitudes toward Evidence-Based Practice were reported;	Participants did not report high barriers to the use of Evidence-Based Practice;
	The perceived lack of knowledge and skills for Evidence-Based Practice (such as literature searching and appraisal) was not considered a significant barrier.	More than 50% frequently use clinical experience and textbooks as their main sources;	The statement with the highest level of agreement was that “keeping up to date with research trends in speech-language pathology is a lifelong professional responsibility”;	The lack of quantity and quality of studies in the area of interest was perceived as an important barrier;
		Less than 10% frequently use research studies, making them the least consulted source;	They tended to disagree with the statement that “research findings are not related to my clinical practice and professionalism.”	Those who used fewer databases or had limited experience with Evidence-Based Practice perceived more barriers;
		Speech-language pathologists with greater clinical experience showed lower use of Evidence-Based Practice.		Speech-language pathologists who had attended conferences in the last two years reported greater barriers than those who had not.
Chu et al.^(23)^, 2021, Japan and Malaysia	Speech-language pathologists with a Master’s or Doctoral degree tend to have greater knowledge of Evidence-Based Practice;	Most speech-language pathologists in Malaysia and Japan base their clinical decisions primarily on personal experience and colleagues’ suggestions rather than on published research;	Speech-language pathologists from Malaysia demonstrated significantly more positive attitudes toward Evidence-Based Practice than those from Japan (p < 0.01);	In both countries, speech-language pathologists with higher levels of education tend to perceive fewer barriers to Evidence-Based Practice;
	Training, particularly at the Master’s level, is suggested to increase self-efficacy and skills in Evidence-Based Practice;	Although not statistically significant, more Malaysian speech-language pathologists reported learning about Evidence-Based Practice through the Internet and workshops than Japanese professionals.	Speech-language pathologists from both countries show positive attitudes toward Evidence-Based Practice, recognizing its potential to improve clinical outcomes and the relevance of research to practice;	Only 27% of speech-language pathologists in Malaysia and 34% in Japan reported a lack of time to practice Evidence-Based Practice, indicating that time is not a significant barrier for them, unlike findings from studies in the United States;
	Essential skills for Evidence-Based Practice include finding valid information, interpreting statistics, and applying information clinically;		Malaysian women working full-time and in government settings reported greater motivation to develop Evidence-Based Practice skills;	Other barriers to the implementation of Evidence-Based Practice include difficulty applying research in practice, limited evidence, workplace constraints, restricted access to resources, unfavorable organizational culture, and lack of knowledge and skills.
	Recent training programs in Malaysia are reinforcing the importance of Evidence-Based Practice skills.		Malaysian speech-language pathologists with a Master’s degree demonstrated a more positive perception of Evidence-Based Practice than those with a bachelor’s degree (p < 0.05).	
Chu et al.^(35)^, 2022, Malaysia and USA	Students from the United States reported greater knowledge of Evidence-Based Practice compared with those from Malaysia;	Research evidence has not been the main influence on clinical decision-making;	Speech-language pathologists in the United States showed interest in additional training and resources to support Evidence-Based Practice;	Difficulty transferring research findings into clinical practice;
	Speech-language pathologists should understand the research process and apply it in clinical practice;	50% of speech-language pathologists use scientific evidence in cases involving tracheostomy;	Most students in the United States considered the application of Evidence-Based Practice necessary and believed that it contributes to better outcomes for clients;	Having limited, conflicting, or irrelevant evidence;
	The main skills cited include formulating clinical questions, searching for relevant evidence, critically appraising the evidence, applying the evidence, and evaluating the outcomes of the process;	Clinical decision-making tends to rely more on personal experience and methods learned during university training than on scientific evidence;	Students from Malaysia and the United States expressed doubts about the validity of basing practice on protocols used by other professionals over time;	Time constraints are reported as a barrier;
	The classroom activity was effective in improving students’ understanding of Evidence-Based Practice and also promoted collaborative learning among peers.	99.6% of speech-language pathologists consider colleagues’ experience a primary and useful clinical source of information;	Both groups, from Malaysia and the United States, were satisfied with the use of Evidence-Based Practice in clinical practice and demonstrated interest and motivation for the activity.	Workplace restrictions and limited access to resources are other barriers.
		Students intend to apply the skills acquired to use Evidence-Based Practice in future clinical practice.		
Cormack, Ailbhe^(36)^, 2010, Texas, EUA	It demonstrated that clinicians who complete more courses in Evidence-Based Practice and research methodology are more likely to implement Evidence-Based Practice.	Clinicians who engage in more research activities and receive greater support in their workplace are more likely to implement Evidence-Based Practice;	Most speech-language pathologists expressed positive views toward Evidence-Based Practice, recognizing its importance for effective and up-to-date clinical practice;	They reported not having sufficient time during working hours to read and apply scientific literature in their clinical cases;
		The American Speech-Language-Hearing Association mandated that speech-language pathologists incorporate Evidence-Based Practice into their clinical decision-making.	Many participants believe that Evidence-Based Practice improves the quality of patient care and should be integrated into daily practice.	Many professionals do not have easy access to scientific databases, specialized journals, or libraries;
				Those with less training in research methodology and statistics showed a lower likelihood of using Evidence-Based Practice;
				They reported difficulty interpreting and applying the results of scientific research in clinical practice.
Cunningham, B.^(37)^,2019, Canada	89% reported that most felt they had the necessary skills to implement Evidence-Based Practice;	After the online learning module, speech-language pathologists reported strong intentions to implement the procedures;	91% stated that the procedures reflect an effective clinical approach;	For Outcome Monitoring: Few barriers; most responses were positive.
	90% reported that they regularly used standardized tools.	The study highlighted the importance of involving clinicians in the development of evidence-based assessments to identify barriers before large-scale implementation. The perceptions analyzed are prior to implementation, which will be evaluated after one year of testing.	87% believe that the process benefits the families and children served.	For the Individual Vulnerability Test: Barriers were reported across all domains: environment, innovation, and skills. Examples include the time required to administer and interpret the tests, lack of materials or administrative support, and the perception that the tests are difficult to integrate into routine practice.
Durieux et al.^(38)^, 2015, Belgium	88.2% of speech-language pathologists in Belgium reported never having heard of Evidence-Based Practice before;	96.9% of speech-language pathologists seek solutions to their problems in practice;	Among the 11.8% who had previously heard of Evidence-Based Practice, 16.3% considered it essential, 24.5% interesting, 22.5% interesting but not feasible, 36.7% reported not having sufficient knowledge to express an opinion, and none considered it uninteresting;	Lack of time (54.2%);
	On a scale from 1 to 10, speech-language pathologists reported mean scores of 6.9 for the ability to search for scientific information, 6.7 for evaluating it, and 7.3 for applying it in practice;	To meet their information needs, most rely on their own resources: personal experience (82.2%), personal libraries (72.4%), and colleagues in the workplace (78.0%);	Speech-language pathologists are generally satisfied with their information-search strategies;	Lack of awareness of field-specific resources (43.9%);
	Higher educational level and more time engaged in continuing education are associated with greater familiarity with Evidence-Based Practice.	On the Internet, professionals prefer to use general search engines: 48.8% for scientific articles and 43.2% for other documents, while only 5.04% use specialized bibliographic databases.	There is expressed interest in training in Evidence-Based Practice (73.0%), information searching (65.2%), and critical appraisal of scientific information (49.9%).	Low proficiency in English (42.4%);
				Difficulty accessing specialized search tools (38.3%);
				Cost of information (37.3%);
				Difficulty selecting relevant documents (35.9%);
				Difficulty evaluating the scientific quality of information (32.5%) and lack of skills in using specialized search tools (27.0%);
				Lack of knowledge or competence related to Evidence-Based Practice (29.5%).
Foster, A^(39)^, 2015, Australia	Speech-language pathologists showed a limited view of Evidence-Based Practice, centered on scientific research while neglecting clinical experience and patients’ preferences;	There is a gap between evidence and practice in the management of Aphasia in the acute phase, with services diverging from best-practice recommendations;	They express a desire to practice Evidence-Based Practice, considering it an “aspirational goal” and a “best practice we should be following”;	Disempowerment emerged as the central theme, arising from a limited view of Evidence-Based Practice, a difficult relationship with research, and the perceived inability to promote change;
	There was a perceived lack of skills to search for research and difficulty understanding recommendations from the scientific literature.	The relationship with research is described as tense, as many perceive that studies do not apply directly to clinical practice;	They place high value on research and Evidence-Based Practice and consider them important;	Lack of resources, staff, and time;
		They show a preference for compiled and pre-appraised research sources, such as clinical guidelines and systematic reviews, rather than original literature.	There is a sense of professional discomfort among speech-language pathologists due to the perception that they are unable to implement evidence-based recommendations;	Organizational priority given to other conditions such as Dysphagia;
			Research is viewed by many as of limited usefulness in clinical practice and disconnected from professional reality. Nevertheless, there is recognition of an ethical imperative to provide evidence-based care.	Perceived scarcity of relevant literature on acute Aphasia;
				Difficulties in searching for, appraising, and applying recommendations;
				Attitudes that discourage seeking literature;
				Lack of supervision and mentorship.
Fulcher-Rood et al.^(40)^, 2020, USA	64% defined Evidence-Based Practice as the use of strategies proven to be effective, with emphasis on research findings;	They use Evidence-Based Practice for treatment decisions but not for assessment;	72% considered research to be the foundation of the Evidence-Based Practice model;	Lack of time was the most frequently cited barrier (64%), caused by high workload, paperwork, and other demands, leading many to carry out Evidence-Based Practice activities outside working hours;
	8% included all three sources of evidence in their definition of Evidence-Based Practice;	They read two research articles per month;	48% considered research valuable mainly for supporting their daily clinical decisions;	16% reported lack of funding;
	They reported difficulty translating research findings into practice;	Among 25 participants, the sources used to search for evidence were general searches (e.g., Google) (56%), the website of the American Speech-Language-Hearing Association (52%), peer-reviewed journals (44%), and speech-language pathology–related websites (32%);	Research was considered less valuable when it did not meet current clinical needs (28%) or when it seemed unfeasible for the work setting, especially in the school context (28%).	16% cited inability to replicate the methods used in research studies;
	36% implement research mainly due to a lack of specific knowledge.	Reasons for not implementing research included the incompatibility of recommendations with clients’ needs (32%) and the difficulty of applying strategies in the school environment due to practical limitations (28%).		Difficulty translating research findings into clinical practice.
Gomez et al.^(41)^, 2019, Australia and New Zealand	Most participants considered that their training had prepared them only partially (58%) or not at all (35%) to treat childhood apraxia of speech (CAS), and only 7% felt adequately prepared;	71% sought information from colleagues;	Many value empirical research evidence;	49% reported being “too busy” to search for or read the literature on Childhood Apraxia of Speech;
	48% reported difficulty interpreting statistical analyses;	68% used their own clinical experience;	35% considered lower levels of evidence to be adequate, as well as higher levels;	Difficulty in “interpreting statistical analyses” was reported by 48%;
	There is a responsibility for academic institutions to prepare students to access and interpret the literature effectively.	62% used journal articles and 52% attended in-person workshops;	21% were uncertain about how to judge the adequacy of empirical research.	46% reported difficulty “accessing articles/reports” related to the treatment of Childhood Apraxia of Speech;
		62% participated in professional development activities related to Childhood Apraxia of Speech.		Perceived limited high-quality evidence for the treatment of Childhood Apraxia of Speech (38%);
				Challenge in understanding the research reported for Childhood Apraxia of Speech (35%).
Greenwell, T. et al.^(22)^, 2021, USA	80% indicated that they understood what constitutes Evidence-Based Practice;	Exposure to Evidence-Based Practice during graduate education and training throughout the career were factors that significantly predicted its use in practice;	89.3% viewed Evidence-Based Practice favorably, indicating that they were “advocates of Evidence-Based Practice”;	54% reported insufficient time to search for and read the literature as the most frequently cited barrier to implementing Evidence-Based Practice;
	81% were confident in determining the optimal intervention when faced with conflicting evidence.	The three most frequently used sources in Evidence-Based Practice were client preferences (mean 4.43), external evidence/research (mean 3.99), and clinical experience (mean 3.87);	80% of respondents indicated that they understood what constitutes Evidence-Based Practice;	43% cited workload as the second most frequently reported barrier;
		65.4% received training in Evidence-Based Practice as part of a graduate-level course.	81% expressed confidence in their ability to determine the optimal intervention when faced with conflicting evidence.	35.37% reported limited access to journal articles at work;
				15.56% identified lack of training as a barrier.
Hegarty, N. et al.^(42)^, 2020, United Kingdom	They reported difficulty “understanding” more complex approaches;	There is a tendency to maintain traditional approaches, while newer or more complex approaches are often neglected;	They perceived the quality of the existing literature as “not robust”;	Insufficient time to search for and read the literature related to Evidence-Based Practice;
	It was suggested that speech-language pathologists develop their own evidence bases (e.g., single-case studies) and improve their skills to co-produce clinically feasible research.	Clinical decisions are based on clinicians’ own experiences and those of their colleagues.	Combining research, clinical experience, and the preferences of the child/parents may result in more effective interventions;	Service-related constraints, such as limited resources, heavy workloads, and difficulty replicating research in real clinical settings;
			They considered that an online, evidence-based resource to support clinical decision-making would be useful.	Difficulties accessing intervention materials play a role in the choice of intervention;
				Levels of confidence (in unfamiliar approaches or in clinical replication);
				Difficulties searching the literature and keeping up to date with current research.
Hoffman, L. M. et al.^(43)^, 2013, USA	Early-career professionals (62%) reported taking more postgraduate courses related to Evidence-Based Practice than experienced professionals (22%);	Most respondents read 0 to 4 articles from the American Speech-Language-Hearing Association per year on topics related to assessment (84%) or intervention (71%);	They demonstrate strong interest in additional training and resources to support Evidence-Based Practice;	One quarter of respondents had no formal training in Evidence-Based Practice;
	The PICO strategy for formulating clinical questions was mentioned as a relevant skill.	The use of online resources from the American Speech-Language-Hearing Association and engagement in Evidence-Based Practice activities were documented as low;	96% were confident in their ability to read and understand research;	91% did not have scheduled time for Evidence-Based Practice activities;
		Previous research has shown that the treatment doses used in studies are generally higher than those feasible in clinical practice.	88% recognized the importance of keeping up to date;	11% worked in school districts with official Evidence-Based Practice guidelines;
			92% trusted the findings of most published research.	60% reported that there were no formally organized Evidence-Based Practice study groups in their district.
Mansuri, B. et al.^(5)^, 2020, Iran	They did not demonstrate comprehensive knowledge of Evidence-Based Practice;	The use of internal evidence was more common than external evidence in clinical practice;	They showed positive and favorable attitudes toward Evidence-Based Practice;	Not reported.
	The mean knowledge score was 3.85 on a scale from 0 to 10;	Frequently used internal evidence includes personal clinical experience (69.3%), client needs and preferences (84.2%), colleagues’ opinions (57.2%), and consultation with specialists (55.5%);	There was high agreement with the statements: “Evidence-Based Practice is the foundation of professional performance” (89.8%), “I am interested in using Evidence-Based Practice in clinical practice” (97.3%), and “I need to use evidence-based treatments” (91.2%);	
	Speech-language pathologists with postgraduate education obtained higher scores (mean of 5.52) than those with only undergraduate education (mean of 2.19);	Among external evidence sources, textbooks are used by more than 70%, online resources such as videos, audio materials, and the internet by nearly 40%, and Telegram/WhatsApp groups by 53.4%;	There were high rates of disagreement with the statements: “Evidence-Based Practice is a waste of time” (93.6%) and “Evidence-Based Practice is a passing trend” (79.5%).	
	It is suggested that speech-language pathologists did not receive adequate training in Evidence-Based Practice during university or educational programs.	Scientific articles are used less frequently in comparison.		
McCurtin, A.^(44)^, 2015, Ireland	Additional qualifications and clinical experience lead to more autonomous and scientifically grounded treatments;	Treatment decisions are mainly based on practice-based evidence and pragmatic considerations;	They defined themselves as dynamic and pragmatic practitioners with an appreciation for the four pillars of Evidence-Based Practice;	There is a lack of high-quality research evidence to guide decisions related to Evidence-Based Practice;
	Lower qualifications are associated with greater use of commercial products rather than evidence-based therapies;	3% reported that research does not influence their decisions;	Factors influencing decision-making did not clearly align with the four pillars of Evidence-Based Practice;	Less experienced clinicians have difficulty applying research in practice;
	Greater experience was correlated with a stronger influence of one’s own clinical experience.	Clinical experience, colleagues’ experience, and professional training influence decision-making.	58% agreed that science should be used to determine whether therapies work;	45% of respondents report lacking time at work to improve treatments;
			48% agreed that clinical experience is the best guide;	For 39% of speech-language pathologists, the limited availability of therapies and techniques is a barrier.
			55% agreed that time may be wasted on unvalidated treatments, and 47% agreed that harm may occur;	
			4% believed that any treatment would work if the therapist believed in it.	
Muttiah, N. et al.^(45)^., 2011, USA	Only one clinician provided a definition of Evidence-Based Practice that included all three aspects; the other clinicians’ definitions tended to focus on only one of these aspects;	Clinical decisions are primarily guided by personal experience and colleagues’ opinions, with less use of scientific literature. Clinicians tend to rely more on their own expertise than on the other pillars of Evidence-Based Practice;	Both groups agreed that Evidence-Based Practice is useful; clinicians view it as a resource to make treatment more ethical and credible, while researchers mainly value the scientific evidence;	Professional time was the most frequently mentioned barrier to the use of Evidence-Based Practice, cited by half of the respondents;
	Some clinicians were not familiar with the use of Evidence-Based Practice or felt uncomfortable with the term.	85% reported having used TONS in the past five years despite the lack of research evidence;	There is disagreement regarding responsibility for effectiveness: researchers avoid using interventions without evidence, whereas some clinicians accept their use if no harm is caused, which contradicts the Code of Ethics of the American Speech-Language-Hearing Association;	Up to 22% of respondents identified the quantity and quality of research, search resources, and lack of knowledge as barriers;
		They stated that they would use Evidence-Based Practice only if it demonstrated results; otherwise, they would return to previous methods.	Both agreed that research should support, but not impose, clinical decision-making.	Clinicians reported difficulty accessing articles due to workload and limited time;
				The lack of evidence on TONS frustrated both groups, attributed to the scarcity of research in child phonology and the high cost of effectiveness studies.
Nail-Chiwetalu et al.^(46)^, 2007, USA	66% interpreted Evidence-Based Practice as basing practices on research literature;	For clinical questions, they mainly relied on colleagues, continuing education, and searches on the open internet;	They considered continuing education activities, personal contacts, and the open internet to be the most useful for their professional needs;	The main barrier reported was the lack of time to search for and read the literature, representing 69% of the barriers to implementing Evidence-Based Practice;
	Only a small proportion reported having received instruction in information literacy from librarians, both formally (9%) and informally (5%);	Few used academic articles to assist with clinical cases;	Evidence-Based Practice is viewed as the integration of research evidence with professional expertise and client values.	Other barriers included difficulty locating appropriate information, using search techniques, and accessing full-text articles.
	Although 60% felt prepared by their graduate education for lifelong learning, their responses emphasized content rather than skills for searching and evaluating information.	67% had consulted websites for clinical information; 59% did not access them routinely for professional purposes, while 41% did;		
		55% felt very successful in analyzing and applying the information they found.		
O'Connor, S. et al.^(24)^, 2009, Ireland	46.9% reported that statistical analysis is not understandable;	There is a prevailing culture of using traditional methods rather than approaches based on Evidence-Based Practice;	Most therapists considered Evidence-Based Practice essential for speech-language pathology practice;	71.9% cited lack of time to read research as the main barrier, while 59.4% mentioned lack of time to implement new ideas related to Evidence-Based Practice;
	37.5% did not feel capable of evaluating the quality of research;	When faced with clinical questions, professionals tend to rely on experience, colleagues’ opinions, older texts, or general websites rather than current literature;	6.3% did not see value in research for practice;	12.5% of respondents or their departments did not subscribe to journals;
	Entry-level therapists showed greater difficulty understanding statistical results (72.8%) compared with senior therapists (42.9%), indicating a possible gap in training or experience related to Evidence-Based Practice.	Many speech-language pathologists seek new practices through professional development courses and conversations with experienced colleagues rather than through scientific articles.	62.5% pointed out methodological inadequacies in research;	A lack of research in specific areas of speech-language pathology was also reported.
			53.1% considered the implications unclear;	
			Half felt that the results were not applicable to their work context;	
			15.6% reported lack of team support for the implementation of Evidence-Based Practice.	
Rojas, C. et al.^(47)^, 2023, Latin America	Most respondents understand Evidence-Based Practice only as scientific publications, ignoring clinical experience and users’ preferences;	Responses regarding the use of Evidence-Based Practice in therapeutic decision-making are varied, with many limiting its use to specific areas, such as Dysphagia, and prioritizing professional experience;	Most respondents believe that Evidence-Based Practice is limited to scientific publications;	The main barrier is the lack of time to search for and read up-to-date scientific literature related to Evidence-Based Practice;
	This view is incorrect, as the American Speech-Language-Hearing Association defines Evidence-Based Practice as the combination of research evidence, clinical expertise, and patient preferences;	Decisions are based on the professional’s experience, traditional practices, and guidelines from ministries of health.	None of the participants consider Evidence-Based Practice fully feasible in their work context, despite recognizing its importance;	Implementation is hindered by unfavorable contexts, short sessions, limited access to information, and language barriers;
	Training of Latin American speech-language pathologists needs to be improved from the undergraduate level to address this misconception.		Although they acknowledge the importance of Evidence-Based Practice, there is resistance to its practical implementation;	Lack of equipment, limited knowledge of scientific searching, low professional engagement, and inflexible organizations;
			There is a perception that international proposals are disconnected from the regional reality.	The belief that valid evidence is lacking in some areas reinforces the continuation of traditional practices.
Sandham, V. et al.^(48)^, 2021, Australia	Lack of skills or resources to provide Evidence-Based Practice was reported by 71% of participants;	87% discuss evidence with colleagues to facilitate Evidence-Based Practice;	Speech-language pathologists hold Evidence-Based Practice in high regard, but the study suggests that there may be room for greater progress in its effective use.	71% reported lack of skills or resources to provide Evidence-Based Practice;
	Speech-language pathologists with little experience in literature searching may have significant difficulty finding and evaluating external evidence.	75% discuss evidence and preferences with clients;		51% cited inadequate resources to implement research evidence as the main barrier;
		64% read reviews and participate in professional events;		36% mentioned conflicts between evidence, client values, and clinical experience;
		61% consult professional guidelines;		26% identified organizational barriers;
		68% apply outcome measures to evaluate their practice and support Evidence-Based Practice.		11% reported lack of consensus among colleagues regarding the implementation of evidence;
				Lack of time was highlighted as an integral factor in the implementation of Evidence-Based Practice.
Sandham, V. et al.^(49)^, 2022, Australia	Clinicians’ capacity in Evidence-Based Practice is essential for providing evidence-based services;	Clinicians seek evidence focused on the individual needs of clients;	Participants feel uncomfortable using evidence of questionable or unknown quality;	Lack of resources, time, and cost hinder access to and use of scientific evidence related to Evidence-Based Practice;
	71% of participants reported lack of skills or resources to provide Evidence-Based Practice;	They participate in educational events and turn to colleagues and social media for quick and accessible information related to Evidence-Based Practice.	The lack of time for Evidence-Based Practice activities is linked to research-search skills and beliefs about its benefits;	Low confidence in search skills and clinical uncertainty are internal barriers;
	Speech-language pathologists with limited experience in literature searching face difficulties finding and evaluating external evidence;		There is a perceived gap in research, especially for specific subpopulations;	Discomfort with self-reflection may hinder the use of Evidence-Based Practice.
	The perceived accessibility of evidence depends on research-search skills and clinical experience to interpret and apply articles.		Some consider research to be poorly applicable or of low clinical value;	
			Negative experiences when accessing evidence reduce motivation to seek original research.	
Souza et al.^(1)^, 2024, Brazil	56.6% indicated that they had learned the foundations of Evidence-Based Practice during their academic training;	82% of participants attend congresses, courses, or scientific updates (40.2% very frequently; 41.8% frequently);	92.6% consider the application of Evidence-Based Practice necessary in speech-language pathology practice;	27.9% indicated that the available time is insufficient to carry out Evidence-Based Practice;
	There are weaknesses in knowledge and in the ability to search for and critically appraise scientific articles;	63.1% have the habit of reading scientific articles (20.5% very frequently; 42.6% frequently);	78.7% believe they need to increase the use of scientific evidence in their daily work;	55.8% agree that there are not enough articles allowing the generalization of findings to the patient population;
	Speech-language pathologists with low proficiency in English have less knowledge and fewer skills related to Evidence-Based Practice;	The most commonly used databases are SciELO (33.6%), PubMed (24.8%), and Google Scholar (23.2%);	Most respondents are uncertain or disagree that Evidence-Based Practice brings positive financial returns;	56.5% acknowledge difficulty applying scientific results to patients with unique characteristics;
	Most respondents disagreed or were undecided about having received formal training in critical appraisal (54.9%), and only 24.6% reported training in search strategies;	None of the participants use the SpeechBITE database, and access to the Cochrane and the evidence map of the American Speech-Language-Hearing Association is rare.	Speech-language pathologists with low proficiency in English tend to believe that Evidence-Based Practice overloads professionals (p = 0.009);	48.4% point to a lack of collective support among colleagues to implement Evidence-Based Practice;
	31.1% felt capable of understanding statistical analysis, while another 31.1% were undecided.		Professionals with more than 10 years since graduation more frequently agree about the lack of evidence for the interventions they use (p = 0.046).	Speech-language pathologists who graduated less than nine years ago perceive time management for Evidence-Based Practice as a greater barrier (p = 0.036);
				The area of child language still lacks sufficient scientific studies for intervention.
Spek et al.^(50)^, 2013, Netherlands	More advanced students showed significantly higher scores in knowledge and skills related to Evidence-Based Practice;	Not reported.	Students from all academic years value Evidence-Based Practice positively but show low self-efficacy regarding their competencies;	The lack of a sense of competence is an important barrier to the use of Evidence-Based Practice among students;
	All groups rated their search skills as inadequate, although second- and third-year students rated these skills as adequate or good on the DMF.		As they progress in their studies, more students perceive Evidence-Based Practice as time-consuming and feel uncertain about their own competence;	As they progress in the program, more students perceive Evidence-Based Practice as time-consuming, which discourages its use;
			Despite this, third-year students consider Evidence-Based Practice more stimulating, with positive scores for task value, although self-efficacy scores remain low.	Additional barriers include difficulty with English when reading articles, scarcity of specific evidence in speech-language pathology, difficulty validating this evidence clinically, limited access to databases, and “numerophobia” (fear of numbers) among students without prior mathematical training.
Thome et al.^(51)^, 2018, USA	13% correctly identified the three components of Evidence-Based Practice;	52% access resources from the American Speech-Language-Hearing Association for professional information;	99% consider Evidence-Based Practice beneficial for providing better treatments;	51% identified lack of time to search for and read the literature as the main barrier to Evidence-Based Practice;
	35% classified meta-analysis as the strongest level of evidence;	21% rely on personal contacts as the second most frequent source;	99% believe it is important to engage in Evidence-Based Practice;	27% cited limited access to information;
	36% indicated randomized clinical trials as the second level of evidence;	29% consider online databases accessed outside libraries as unlikely sources of information;	62% find it difficult to engage in Evidence-Based Practice;	24% mentioned the lack of relevant information for their clinical cases;
	61% correctly classified expert reports and clinical experience as the weakest level;	University, public, or medical libraries (35%) and non-academic websites (43%) are among the least accessed sources;	Most respondents consider that their employers are familiar with Evidence-Based Practice (33% “very knowledgeable,” 45% “somewhat knowledgeable”) and encourage its use (43% “strongly agree,” 36% “somewhat agree”);	Only 15% reported that their employer provides time for Evidence-Based Practice updates;
	23% reported feeling “very knowledgeable” about using academic and medical libraries, and 35% reported the same regarding online databases outside libraries;	The study highlights that self-reported use of Evidence-Based Practice does not always reflect actual adherence to Evidence-Based Practice guidelines.	The most useful sources were resources from the American Speech-Language-Hearing Association (46%) and personal contacts (27%);	Only 24% stated that their employer provides access to paid content (databases or journals).
	49% reported feeling “very confident” and 45% “somewhat confident” in reading and interpreting scientific studies.		The least useful sources were non-academic websites (35%) and university/public/medical libraries (36%).	
Tohidast et al.^(52)^, 2017, Iran	46.4% received formal education on Evidence-Based Practice at university, while about half did not have this type of training;	53% read between 2 and 5 articles in the last month, while only 3.2% read more than 15;	Speech-language pathologists in Iran showed a positive attitude toward Evidence-Based Practice;	62% identified lack of time as the main barrier to implementing Evidence-Based Practice;
	63% reported confidence in their searching skills and 67% in critical appraisal skills, although many do not understand technical terms;	38.6% searched databases fewer than two times in the month;	They agreed or strongly agreed that Evidence-Based Practice is necessary for speech-language pathology (96.8%) and that it improves the quality of patient care (96.1%);	44.7% reported lack of research skills, and 40.7% highlighted difficulty applying research findings to patients with unique characteristics;
	Formal training increased confidence in Evidence-Based Practice skills, and women reported greater confidence than men in implementing it.	81% used literature for clinical decisions fewer than six times in the month related to Evidence-Based Practice;	81.4% of respondents disagreed or strongly disagreed that adopting Evidence-Based Practice imposes an unreasonable demand on professionals;	Lack of interest was considered the least relevant barrier.
		52% had access to printed journals;	73.6% disagreed or strongly disagreed that Evidence-Based Practice does not consider patient preferences.	
		45.7% had access to databases at work, while 87% had access at home or another location.		
Tohidast et al.^(53)^, 2021, Iran	Not reported.	Not reported.	Some negative personal attitudes function as barriers, such as feeling scientifically competent enough or believing that current knowledge is sufficient without seeking further updates related to Evidence-Based Practice;	Lack of knowledge and skills in Evidence-Based Practice;
			Excessive pride leads to the belief that current techniques and methods are sufficient, without the need to learn new ones;	Negative personal attitudes (overconfidence in current knowledge and resistance to learning);
			Social culture influences opinions, including a “culture of comfort” that prefers passive forms of learning, such as lectures and workshops, while avoiding individual effort;	Lack of time to search for and apply Evidence-Based Practice;
			There is also a clinician-centered culture in which patients and families place excessive trust in the professional’s decisions and avoid active participation in clinical decision-making, which contradicts the principles of Evidence-Based Practice.	Insufficient academic education on Evidence-Based Practice and scientific research;
				Inadequate infrastructure in clinics for implementing Evidence-Based Practice;
				Financial pressures to prescribe more therapy sessions, which may contradict Evidence-Based Practice;
				Scarcity of evidence and guidelines specific to the regional context;
				Low public awareness of Evidence-Based Practice, leading to family resistance;
				Financial limitations of families that hinder access to evidence-based treatments.
Vallino-Napoli, L. D. et al.^(54)^, 2004, Australia	Most clinicians were familiar with Evidence-Based Practice and had a general understanding of its meaning;	All respondents had participated in continuing education in the previous year and had access to the Internet, but accessed these sources infrequently;	Most clinicians had heard of Evidence-Based Practice and highly valued research;	Lack of time for reading and searching the literature is the main barrier to Evidence-Based Practice;
	94% associated Evidence-Based Practice with the application of findings from clinically relevant studies;	53% of clinicians used computer databases to search the literature related to Evidence-Based Practice;	75% agreed that “All speech-language pathologists should take a mandatory course in research methodology”;	93% agree that time should be allocated for these activities, but 69% reported not having dedicated time at work;
	51% included the application of clinical skills and experience in their definition of Evidence-Based Practice;	90% used professional journals as a source of evidence;	66% agreed that “Research helps me achieve what I want as a speech-language pathologist”;	Limited frequency of access to evidence sources is the second barrier;
	28% considered it important to integrate patients’ opinions into clinical decision-making;	72% relied on established clinical guidelines;	44% agreed that “It is difficult to apply research in practice”;	Difficulty understanding statistics in studies (78% in a cited study);
	3% did not know what Evidence-Based Practice meant;	The most frequently consulted sources for patient management were lectures (89%), consultation with a local specialist (86%), journal articles (70%), the Internet (64%), special interest groups (53%), and journal clubs (51%).	26% agreed that “Basing practice on research findings would cost too much time and money”;	Unclear implications for practice (66% in the cited study);
	Clinicians with up to 10 years of practice more frequently used research findings to guide their practice, possibly due to changes in the curriculum.		14% agreed that “Most speech-language pathologists are not interested in implementing research findings.”	Literature dispersed across many journals;
				Access to computer-based resources may be time-consuming and inefficient, which discourages busy clinicians.
Zipoli Jr, R. P. et al.^(25)^, 2005, USA	13% of speech-language pathologists reported lack of knowledge and skills as a barrier to Evidence-Based Practice, indicating that most feel competent;	The most frequently used sources were personal clinical experience (99.6%) and colleagues’ opinions (78.7%);	The most frequently used sources were personal clinical experience (99.6%) and colleagues’ opinions (78.7%);	Lack of professional time was the most significant barrier, with 50.0% of respondents disagreeing or strongly disagreeing that they had time to engage in Evidence-Based Practice;
	Graduate education, which may include training in statistics and research methods, contributes to this competence;	The least used sources were case studies (15.9%), videos or audiotapes (17.3%), and research studies (17.7%);	The least used sources were case studies (15.9%), videos or audiotapes (17.3%), and research studies (17.7%);	Barriers related to knowledge and skills (13.0%), resources (17.6%), and the quantity/quality of research (21.8%) were perceived to a lesser extent by the speech-language pathologists in this study.
	Exposure to research and Evidence-Based Practice during graduate training and the Clinical Fellowship Year (CFY) is an important predictor of positive attitudes toward Evidence-Based Practice.	There was a significant decrease in exposure to Evidence-Based Practice and research from the graduate period (mean 2.19) to the Clinical Fellowship Year (CFY) (mean 3.23), with higher values indicating lower exposure.	There was a significant decrease in exposure to Evidence-Based Practice and research from the graduate period (mean 2.19) to the Clinical Fellowship Year (CFY) (mean 3.23), with higher values indicating lower exposure.	

**Caption:** EBP: Evidence-Based Practice; ASD: Autism Spectrum Disorder; ASHA: American Speech-Language-Hearing Association; CAS: Childhood Apraxia of Speech; NSOMTs: Non-Speech Oral Motor Treatments; CFY: Clinical Fellowship Year
