# Supplementary material for: Opinions of speech-language-hearing pathologists and students on evidence-based practice: a systematic review
Source: Codas. 2026 Jul 6;38(4):e20250300. doi: 10.1590/2317-1782/e20250300en (PMC13343793; doi:10.1590/2317-1782/e20250300en)
Supplement: Translation

# Translation: Opiniões de fonoaudiólogos e estudantes sobre a prática baseada em evidências: uma revisão sistemática

**DOI:** 10.1590/2317-1782/e20250300pt

**Authors:** Samara Fernandes da Silva Souza, Allya Francisca Marques Borges, Ramon Cipriano Pacheco de Araújo, Denise da Silva Medeiros, Suzanne Bettega Almeida, Cristiano Miranda de Araujo, Hipólito Virgílio Magalhães, Karinna Veríssimo Meira Taveira

**Affiliations:** 1 Departamento de Fonoaudiologia, Universidade Federal do Rio Grande do Norte – UFRN - Natal (RN), Brasil.; 2 Grupo de Pesquisa Estudos em Motricidade Orofacial e Disfagia Orofaríngea, Universidade Federal do Rio Grande do Norte – UFRN - Natal (RN), Brasil.; 3 Programa de Pós-graduação em Saúde da Comunicação Humana, Universidade Federal de Pernambuco – UFPE – Recife (PE), Brasil.; 4 Núcleo de Estudos Avançados em Revisão Sistemática e Meta-análise – NARSM, Programa de Pós-graduação em Distúrbios da Comunicação, Universidade Tuiuti do Paraná – UTP - Curitiba (PR), Brasil.; 5 Programa de Pós-graduação em Odontologia, Pontifícia Universidade Católica do Paraná - PUC - Curitiba (PR), Brasil.; 6 Núcleo de Estudos Avançados em Revisão Sistemática e Meta-análise – NARSM, Programa Associado de Pós-graduação em Fonoaudiologia, Departamento de Morfologia, Centro de Biociências, Universidade Federal do Rio Grande do Norte – UFRN - Natal (RN), Brasil.

**Keywords:** Prática Baseada em Evidências, Fonoaudiologia, Atitude, Conhecimento, Revisão Sistemática

## Abstract

**Objetivo:**

Sintetizar evidências sobre os conhecimentos, habilidades, atitudes, comportamentos e barreiras relatadas por fonoaudiólogos e estudantes de Fonoaudiologia em relação à prática baseada em evidências (PBE).

**Estratégia de pesquisa:**

Realizou-se uma revisão sistemática com buscas nas bases PubMed/MEDLINE, Scopus, Web of Science, Embase, LILACS, SciELO, LIVIVO e literatura cinzenta.

**Critérios de seleção:**

Foram incluídos estudos observacionais que investigaram aspectos relacionados à PBE entre profissionais e graduandos da Fonoaudiologia, com dados coletados por meio de questionários.

**Análise dos dados:**

A extração dos dados foi realizada por dois revisores, de forma independente, e a síntese das informações foi apoiada pela ferramenta de inteligência artificial NotebookLM, com posterior verificação manual. O risco de viés foi avaliado considerando representatividade da amostra, taxa de resposta, precisão dos dados, evidência de cálculo amostral e qualidade do instrumento utilizado.

**Resultados:**

Foram incluídos 31 estudos publicados entre 2004 e 2024, com amostras variando de 9 a 2.762 participantes. O risco de viés variou de 1 a 4 em uma escala de 0 a 6. Os estudos evidenciam atitudes geralmente favoráveis à PBE, mas indicam limitações importantes nas habilidades de busca em bases de dados, leitura crítica e aplicação das evidências. A prática clínica ainda se baseia fortemente na experiência pessoal, com uso restrito da literatura científica. As principais barreiras relatadas foram falta de tempo, acesso limitado às evidências, escassez de evidências aplicáveis e lacunas na formação.

**Conclusão:**

Apesar das atitudes positivas, a adoção da PBE na Fonoaudiologia ainda é limitada, exigindo estratégias educacionais e institucionais para fortalecer sua implementação.

## INTRODUÇÃO

A prática baseada em evidências (PBE) refere-se a um conjunto de critérios utilizados para avaliar a evidência científica. Diversos campos da saúde, incluindo medicina, fisioterapia, enfermagem, fonoaudiologia, terapia ocupacional, nutrição e odontologia, adotaram a PBE^(1-5)^. O objetivo da PBE é utilizar as melhores evidências de pesquisa disponíveis para informar decisões clínicas sobre cuidados de saúde, garantindo que os pacientes recebam tratamentos atualizados, seguros e eficazes. A PBE é a integração de três componentes: a experiência clínica do profissional, evidência (externa e interna) e a perspectivas do cliente e/ou paciente e cuidador^(6)^. Nesse sentido, a implementação da PBE é um importante recurso clínico fonoaudiológico, pois, além de discutir e implementar as evidências existentes, constantemente se reflete sobre as necessidades da área e insuficiência das evidências para suportar algumas estratégias de gestão da prática clínica, seja com relação à qualidade e/ ou quantidade de evidências^(7)^.

Embora os conceitos da PBE estejam bem definidos, sua implementação enfrenta diversos desafios^(8)^. Esses desafios estão intimamente relacionados com a situação atual das políticas de saúde, a complexidade da prática fonoaudiológica, o acesso aos estudos e programas de educação continuada. Estudos anteriores em diferentes profissões de saúde identificaram várias barreiras, como a falta de tempo^(9-12)^, a falta de acesso a artigos em texto completo^(13,14)^ e a falta de habilidades para encontrar e compreender os estudos^(11,14-16)^. A incapacidade dos profissionais de saúde para compreender e selecionar estudos de alta qualidade tem sido atribuída ao treinamento inadequado em PBE, devido à grande variabilidade dos métodos de ensino durante a formação universitária^(16)^. Outras barreiras incluem a qualidade questionável dos estudos^(15,17,18)^ e os resultados conflitantes de diferentes estudos sobre o mesmo tema^(10,18)^, a falta de domínio das estratégias de busca e avaliação crítica dos artigos científicos^(1)^. Alguns estudos apresentam características que não representam a prática clínica real, dificultando sua aplicação^(11,19,20)^. Outro fator que pode interferir na aplicação da PBE é o idioma de publicação, uma vez que a maioria dos estudos é publicada em inglês^(21)^, o que pode dificultar seu uso por leitores não proficientes nesse idioma.

Apesar de possuírem algum embasamento teórico, os fonoaudiólogos que atuam com transtornos da linguagem e outras áreas da fonoaudiologia em nível internacional reconhecem a insuficiência de tempo, a carga de trabalho extensa, a escassez de pesquisas na área, a qualidade das evidências disponíveis e a falta de recursos no ambiente de trabalho como os principais obstáculos para a execução da PBE^(2,22-24)^. O treinamento formal sobre as bases da PBE durante a graduação ou em formação continuada aparece como um forte preditor para a execução dessa prática na atuação clínica^(22,25,26)^.

Dessa forma, considera-se importante realizar uma revisão sistemática para melhor informar os profissionais sobre essas características e identificar as dificuldades mais relevantes enfrentadas pelos fonoaudiólogos em relação à PBE. Assim, o objetivo do presente estudo foi revisar sistematicamente as evidências sobre o conhecimento, habilidades, comportamentos, opiniões e barreiras enfrentadas pelos fonoaudiólogos em relação à prática baseada em evidências.

## MÉTODO

### Protocolo e registro

O protocolo desta revisão sistemática foi registrado no PROSPERO® (CRD42025647859), (International Prospective Register of Systematic Reviews - Centre for Reviews and Dissemination, University of York) e conduzido de acordo com a checklist PRISMA-2020 (Preferred Reporting Items for Systematic Reviews and Meta-Analysis)^(27)^.

### Pergunta de pesquisa

O acrônimo “PECOS” (Participantes, Exposição, Controle, Resultados e Desenho do Estudo) orientou a construção da questão de pesquisa. A Figura 1 descreve a estruturação do PECOS utilizado para abordar a seguinte questão focada: “Quais os conhecimentos, habilidades, comportamentos, opiniões e barreiras enfrentadas pelos fonoaudiólogos em relação à PBE?”

**Figura 1:** O acrônimo “PECOS” como guia para identificar a questão de pesquisa

### Critérios de elegibilidade

Foram incluídos estudos observacionais de coorte e de corte transversal que investigaram as características da PBE entre graduandos e profissionais de Fonoaudiologia, com foco em conhecimentos, habilidades, comportamentos, opiniões e barreiras, avaliados por meio de questionários. Não houve exclusão de estudos baseado no idioma, sexo e tempo de publicação.

Foram excluídos estudos que:

1. Não envolvam estudantes ou profissionais da fonoaudiologia.
2. Não relatem conhecimentos, habilidades, comportamento, opiniões e barreiras em fonoaudiologia em relação à PBE.
3. Revisões, cartas ao editor, relatos de casos, séries de casos, opiniões de especialistas e diretrizes.

### Fontes de informação e estratégia de busca

Truncamentos e combinações de palavras foram adaptados para cada estratégia de busca nas seguintes bases de dados: Embase, Literatura Latino-Americana e do Caribe em Ciências da Saúde (LILACS), LIVIVO, PubMed (incluindo MedLine), Scientific Electronic Library Online (SciELO), Scopus e Web of Science. Foram utilizados descritores, palavras-chave e termos livres relacionados a "Evidence-Based Practice", "Attitude", "Opinions", "Knowledge", "Barrier", "Ability" e "Speech-Language Pathologists", combinados com operadores booleanos (OR e AND). A busca completa foi realizada em 19 de novembro de 2024 e pode ser consultada Apêndice 1. Além disso, foi realizada uma busca na literatura cinzenta por meio do Google Scholar e do ProQuest Dissertations & Theses. Buscas manuais também foram conduzidas nas listas de referências dos estudos incluídos, e especialistas foram consultados para identificar estudos adicionais. O software EndNote® X7 (Thomson Reuters, Philadelphia, PA). Foi utilizado para gerenciar as referências e eliminar duplicatas. Para a seleção dos estudos, utilizou-se a plataforma Rayyan®, com revisão cega por dois avaliadores e a atuação de um terceiro revisor como moderador.

### Seleção dos estudos

Dois autores (SFSS e DSM) da revisão realizaram, de forma independente, a triagem de títulos e resumos identificados pela estratégia de busca, com base nos critérios de elegibilidade (Fase 1). Em seguida, avaliaram também de forma independente os textos completos dos estudos, considerando os critérios de inclusão e exclusão (Fase 2). As discordâncias foram resolvidas por meio de discussão com um terceiro autor e posterior consenso entre os três revisores (KVMT).

### Processo de coleta de dados e lista de dados

A extração dos dados foi realizada com o apoio da ferramenta de inteligência artificial NotebookLM^(28)^, que auxiliou na leitura, categorização e síntese dos textos científicos incluídos. De cada estudo, foram extraídas as seguintes informações: autor, ano de publicação, país de realização, características da amostra, número de participantes (incluindo a taxa de resposta, quando informada), instrumento utilizado e principais aspectos da PBE investigados. As informações processadas pela ferramenta foram verificadas por um dos revisores (SFSS), e eventuais dúvidas ou discrepâncias foram discutidas e resolvidas em conjunto com um segundo revisor (KVMT), com experiência prévia na temática da revisão.

### Risco de viés em cada estudo

O risco de viés foi avaliado utilizando os critérios previamente descritos na literatura^(29,30)^, que consideram a representatividade da amostra, a taxa de resposta, a precisão dos dados, a apresentação de cálculo de poder amostral e o instrumento utilizado. Cada estudo recebeu uma pontuação em uma escala de 0 a 6 pontos, na qual escores mais altos indicam menor risco de viés. A avaliação foi conduzida de forma independente por dois revisores (SFSS e DSM), e eventuais discrepâncias foram resolvidas com a mediação de um terceiro revisor (KVMT).

### Avaliação do viés de relato

Na impossibilidade de avaliar o viés de publicação através de análise gráfica (n < 10), uma ampla estratégia de busca foi realizada, em diversas bases de dados e literatura cinzenta, incluindo uma base com idioma diferente do inglês.

## RESULTADOS

### Seleção dos estudos

A estratégia de busca recuperou 1.950 registros provenientes das bases de dados científicas e da literatura cinzenta. Na triagem inicial (fase 1), com base na leitura dos títulos e resumos, foram excluídos 1.896 registros, resultando 54 estudos potencialmente elegíveis para leitura do texto completo. Desses, um artigo não pôde ser recuperado, sendo, portanto, não avaliado. Assim, 53 estudos tiveram seus textos completos analisados (fase 2), dos quais 22 foram excluídos por não atenderem aos critérios de elegibilidade previamente estabelecidos (Apêndice 2). Ao final do processo de seleção, 31 estudos foram incluídos na síntese qualitativa (Figura 2). Não foram identificados estudos adicionais nas referências dos trabalhos selecionados.

**Figura 2:** Diagrama de fluxo da seleção dos estudos/fonte de evidências

*Fonte:* Page et al.^(27)^. Para obter mais informações, visite: http://www.prisma-statement.org/

### Características dos estudos

A Tabela 1 apresenta a descrição das características dos estudos incluídos. Dos 31 estudos analisados, os participantes eram provenientes de países distintos, sendo eles: Arábia Saudita, Malásia, Japão, Coreia do Sul, Estados Unidos, Canadá, Bélgica, Austrália, Nova Zelândia, Reino Unido, Irlanda, Irã, Brasil, Argentina, Paraguai, Chile, Peru, Colômbia, Uruguai, Costa Rica e Países Baixos. As publicações datam do período entre 2004 e 2024. O tamanho das amostras variou entre os estudos, com número de participantes entre 9^(47)^ e 2.762^(43)^ indivíduos.

**Tabela 1:** Caracterização dos estudos incluídos na análise (n=31)

**Nome, Data, País**	**Dados da amostra**	**Número de Participantes (Taxa de Resposta, %)**	**Instrumento Utilizado**	**Aspectos da PBE (Prática Baseada em Evidências)**
Alaidary, Abdulsalam et al.^(2)^, 2020, Arabia Saudita	Fonoaudiólogos	n=65* (73,8) *n= 48 participou	Questionário online	Atitudes favoráveis a PBE, conhecimento, comportamento, opiniões, barreiras e habilidades
Bennett et al.^(31)^, 2019, Austrália	Fonoaudiólogos	n= 145 (NR)	Questionário online com respostas abertas via Qualtrics	Imprescindibilidade de uma equipe multiprofissional, comportamentos, opiniões e barreiras
Chan A. K. et al.^(32)^, 2013, Austrália	Fonoaudiólogos	n= 58 (NR)	Questionário online com perguntas em Likert via Qualtrics	Conhecimento, habilidades, comportamentos, opiniões e barreiras
Cheung et al.^(33)^, 2013, Austrália	Fonoaudiólogos	n= 772* (13,6) *n= 105 participou	Questionário online	PBE se faz necessária, conhecimento, habilidades, comportamentos, opiniões e barreiras
Choi et al.^(34)^, 2015, Coréia do Sul	Fonoaudiólogos	n= 274* (85) *n= 234 participou	Questionário online	Atitude geralmente positiva em relação a PBE, conhecimento, habilidades, comportamentos, opiniões e barreiras
Chu et al.^(23)^, 2021, Japão e Malásia	Fonoaudiólogos	n= 150* (NR) *n= 145 participou	Questionário online com perguntas em Likert	Recursos disponíveis, variáveis demográficas, comparação transcultural, conhecimento, habilidades, comportamento, opiniões e barreiras
Chu et al.^(35)^, 2022, Malásia e EUA	Estudantes de Fonoaudiologia	n= 69 (NR)	Atividade prática e perguntas em Likert	Comparação transcultural, relevância da formação universitária, conhecimento, habilidades, comportamentos, opiniões e barreiras.
Cormack, Ailbhe^(36)^, 2010, EUA	Fonoaudiólogos	n= 85 (NR)	Questionário estruturado	Conhecimento, habilidades, comportamentos, opiniões e barreiras
Cunningham, B.^(37)^, 2019, Canadá	Fonoaudiólogos	First survey n=56* (96) *n=54 participou Second survey n=28* (90) *n=25 participou	Módulo online educativo + dois questionários com perguntas em Likert	Conhecimento, habilidade, comportamento, opiniões e barreiras
Durieux et al.^(38)^, 2015, Bélgica	Fonoaudiólogos	n= 2068*; (20) *n= 415 participou	Questionário online	Fatores que influenciam a implementação da PBE, conhecimento, habilidade, comportamento, opiniões e barreiras
Foster, A.^(39)^, 2015, Austrália	Fonoaudiólogos	n= 14 (NR)	Entrevistas semiestruturadas	Facilitadores da PBE, conhecimento, habilidade, comportamento, opiniões e barreiras
Fulcher-Rood et al..^(40)^, 2020, EUA	Fonoaudiólogos	n= 25 (NR)	Entrevistas telefônicas semiestruturadas	Lacuna entre pesquisa e prática, conhecimento, habilidade, comportamento, opiniões e barreiras
Gomez et al.^(41)^, 2019, Austrália e Nova Zelândia	Fonoaudiólogos	n= 138* (79) *n= 109 participou	Questionário online com perguntas em Likert	Conhecimento, habilidade, comportamento, opiniões e barreiras
Greenwell, T. et al.^(22)^, 2021, EUA	Fonoaudiólogos	n= 324*; (97,8) *n= 317 participou	Questionário online	Impacto do treinamento em PBE, conhecimento, habilidade, comportamento, opiniões e barreiras e a relação entre essas barreiras
Hegarty, N. et al.^(42)^, 2020, Reino Unido	Fonoaudiólogos	n= 21 (NR)	Entrevistas e grupos focais	Fatores que influenciam a decisão clínica, conhecimento, habilidade, comportamento, opiniões e barreiras
Hoffman, L. M. et al.^(43)^, 2013, EUA	Fonoaudiólogos	n= 2.762*; (87) *n= 2.394 participou	Questionário online	Recursos Disponíveis para apoiar a PBE, conhecimento, habilidade, comportamento, opiniões e barreiras
Mansuri, B. et al.^(5)^, 2020, Irã	Fonoaudiólogos	n= 600*; (70) *n=411 participou	Questionário auto aplicado (SLP-EBPQ)	Relação do nível educacional com a PBE, Conhecimento, habilidade, comportamento e opiniões.
McCurtin, A.^(44)^, 2015, Irlanda	Fonoaudiólogos	n= 249 (NR)	Questionário online	Conhecimento, habilidade, comportamento, opiniões e barreiras
Muttiah, N. et al.^(45)^, 2011, EUA	Fonoaudiólogos	n= 22 (NR)	Entrevistas qualitativas presenciais e via ligação telefônica	Conhecimento, habilidade, comportamento, opiniões e barreiras
Nail-Chiwetalu et al.^(46)^, 2007, EUA	Fonoaudiólogos	n= 1.000*; (21) *n= 208 participou	Questionário online	Conhecimento, habilidade, comportamento, opiniões e barreiras
O'Connor, S. et al.^(24)^, 2009, Irlanda	Fonoaudiólogos	n= 39*; (82,1) *n= 32 participou	Questionário via correio	Conhecimento, habilidade, comportamento, opiniões e barreiras
Rojas, C. et al.^(47)^, 2023, América Latina	Fonoaudiólogos	n= 22*; (41) *n= 9 participou	Entrevistas semiestruturadas via correio eletrônico	Realidades regionais, conhecimento, comportamento, opiniões e barreiras
Sandham, V. et al.^(48)^, 2021, Austrália	Fonoaudiólogos	n= 198*; (55,1%) *n= 109 participou	Questionário online	Conhecimento, habilidade, comportamento, opiniões e barreiras
Sandham, V. et al.^(49)^, 2022, Austrália	Fonoaudiólogos	n= 15 (NR)	Grupos focais	Modelagem e prática reflexiva para o uso no contexto clínico, conhecimento, habilidade, comportamento, opiniões e barreiras
Souza et al.^(1)^, 2024, Brasil	Fonoaudiólogos	*n= 122 (NR)	Questionário online	Conhecimento, habilidade, comportamentos, opiniões e barreiras
Spek et al.^(50)^, 2013, Países Baixos	Estudantes de Fonoaudiologia	n= 182*; (82%) *n= 149 participou	Questionário presencial (DMF)	Auto eficácia e valor de tarefa, conhecimento, habilidade, opiniões e barreiras
Thome et al.^(51)^, 2018, EUA	Fonoaudiólogos	n= 285*; (61,75) *n= 176 participou	Questionário online	Conhecimentos, habilidades, comportamentos, opiniões e barreiras
Tohidast et al.^(52)^, 2017, Irã	Fonoaudiólogos	n= 200*; (63,5) *n= 127 participou	Questionário online	Conhecimentos, habilidades, comportamentos, opiniões e barreiras
Tohidast et al.^(53)^, 2021, Irã	Fonoaudiólogos	n= 30*; (46,7) *n= 14 participou	Entrevistas semiestruturadas	Opiniões e barreiras
Vallino-Napoli, L. D. et al.^(54)^, 2004, Austrália	Fonoaudiólogos	n= 697* (54,2) ^*^n= 378 participou	Questionário via correio	Conhecimentos, habilidades, comportamentos, opiniões e barreiras
Zipoli Jr, R. P. et al.^(25)^, 2005, EUA	Fonoaudiólogos	n= 488^*^; (49,2) *n= 240 participou	Questionário via correio	Conhecimentos, habilidades, comportamentos, opiniões e barreiras

* n: número de respostas

**Legenda:** NR: Não reportado; n: Número de convites enviados; SLP-EBPQ: Speech and Language Pathology Evidence-Based Practice questionnaire; DMF: Dutch Modified Fresno

Os participantes dos estudos incluídos eram majoritariamente fonoaudiólogos, atuando em uma variedade de ambientes profissionais. Muitos trabalhavam em hospitais públicos e privados^(41)^ incluindo unidades de AVC agudo^(39)^, hospitais gerais^(45)^, instalações hospitalares e ambientes de cuidados agudos^(24)^. Outros estavam inseridos em escolas públicas e privadas^(46)^, abrangendo escolas de ensino fundamental, médio ou superior^(25)^ escolas especiais^(41)^ e contextos escolares específicos (school-based)^(43)^. Também foram identificados profissionais em clínicas e consultórios privados^(46)^, como em prática autônoma, clínicas universitárias e serviços prestados no domicílio do cliente^(41)^. Havia ainda participantes atuando em contextos comunitários, como centros de saúde comunitários^(41)^, bem como em ambientes de cuidado geriátrico e residencial^(31)^, como casas de repouso^(46)^. Alguns trabalhavam em organizações governamentais e serviços públicos^(33)^, incluindo programas como o Infant Hearing Program no Canadá^(37)^ e estruturas de saúde e assistência social como os HSCTs na Irlanda do Norte^(42)^. Além disso, foram citadas Organizações Não Governamentais (ONGs)^(41)^ e agências voluntárias voltadas ao atendimento de pessoas com deficiência intelectual e transtorno do espectro autista (TEA)^(24)^, bem como centros de reabilitação^(46)^. Por fim, alguns profissionais atuavam em outros contextos, como serviços de saúde domiciliar, programas de intervenção precoce, como subcontratados independentes^(46)^, ou simplesmente referidos como "unidade" ou "departamento" onde trabalhavam^(53)^.

Diversos estudos incluídos nesta revisão basearam o desenvolvimento de seus instrumentos de coleta de dados em pesquisas anteriores, sobretudo no contexto da PBE na Fonoaudiologia. Os questionários analisados, apesar de variações metodológicas, compartilham temáticas recorrentes, como atitudes, habilidades, fontes utilizadas e barreiras percebidas à implementação da PBE.

Muitos dos questionários foram adaptados, como os desenvolvidos por Zipoli e Kennedy, Jette et al., Nail-Chiwetalu et al., Vallino-Napoli e Reilly e Funk et al. (1991), frequentemente utilizados em estudos com profissionais da Fonoaudiologia e de áreas afins. Esses instrumentos serviram de base para pesquisas em diferentes contextos, como nos estudos de Alhaidary et al., Chu et al. e Greenwell et al., que adaptaram itens específicos conforme a realidade local e os objetivos investigativos.

Alguns estudos optaram por desenvolver seus próprios instrumentos com base em modelos anteriores, a exemplo de Cheung et al., que utilizaram um questionário online adaptado da Fonoaudiologia para o contexto do autismo, e Souza et al., que adaptaram instrumentos da Fisioterapia para a realidade brasileira. Mesmo em estudos qualitativos, como o de Sandham et al., as entrevistas foram guiadas por roteiros baseados em dados quantitativos prévios, demonstrando integração entre abordagens metodológicas.

### Risco de viés nos estudos

A pontuação atribuída aos estudos incluídos variou entre 1 e 4 pontos, em uma escala de 0 a 6, conforme os critérios utilizados para avaliação do risco de viés. A maioria dos estudos obteve entre 1 e 3 pontos, enquanto dois estudos alcançaram 4 pontos^(24,49)^. Os critérios mais frequentemente atendidos foram a coleta de dados com foco em aspectos da PBE, como atitudes, habilidades, opiniões, barreiras e comportamentos, seguidos pela descrição da amostra e da taxa de resposta. Por outro lado, os critérios menos frequentemente relatados incluíram a ausência de informações sobre os não respondentes, a falta de validação dos instrumentos utilizados e a ausência de descrição do cálculo de poder amostral. A Figura 3 apresenta a pontuação individual dos estudos conforme os critérios avaliados.

**Figura 3:** Avaliação de risco de viés dos estudos incluídos

### Resultados individuais dos estudos

#### Conhecimento da PBE

Os estudos analisados revelam que fonoaudiólogos e estudantes frequentemente apresentam uma compreensão parcial da PBE, muitas vezes reduzida à utilização exclusiva de resultados de pesquisa científica, com pouca valorização da experiência clínica e das preferências dos pacientes^(22,40,47)^. Apenas uma minoria demonstra reconhecer os três pilares que compõem a PBE. Em diferentes levantamentos, apenas 8%, 14% e 28% dos participantes referiram os três componentes corretamente^(40,51,54)^. Além disso, alguns profissionais acreditam, de forma equivocada, que a PBE exige o uso da “última evidência publicada”, e não necessariamente da melhor evidência disponível^(47)^.

Profissionais com formação de pós-graduação tendem a apresentar melhor desempenho em testes sobre PBE e maior familiaridade com seus conceitos centrais^(5,23)^. No entanto, mesmo entre esses profissionais, são evidentes lacunas em habilidades técnicas fundamentais, como a interpretação estatística, a avaliação crítica da literatura e a busca por estudos relevantes. No Brasil, apenas 31,1% dos participantes declararam compreender análises estatísticas, e a maioria informou não ter recebido treinamento formal para avaliação crítica de estudos científicos^(1)^. Essas dificuldades são potencializadas pela barreira linguística, já que a maioria das publicações relevantes está disponível apenas em inglês, idioma que ainda representa um entrave para muitos profissionais^(1,20)^.

A Figura 4 ilustra os desfechos mais frequentemente relatados em relação aos países, e o Apêndice 3 apresenta todos os aspectos incluídos em cada estudo. Em relação à distribuição dos aspectos da PBE investigados nos estudos, observou-se que os domínios mais frequentemente analisados foram conhecimento, opiniões e barreiras, cada um relatado em estudos de 14 países. O domínio habilidades foi abordado em 10 países, enquanto comportamento foi o menos investigado, presente em estudos de 9 países. Destaca-se que Brasil, Estados Unidos e Austrália concentraram a maior variedade de domínios avaliados, enquanto países como Irlanda e Irã focalizaram principalmente em conhecimento e barreiras. Esses achados evidenciam a heterogeneidade conceitual e metodológica entre os estudos internacionais sobre PBE e reforçam a importância de mapeamentos amplos, como o presente, para compreender a diversidade de abordagens, contextos e níveis de implementação dessa prática entre fonoaudiólogos.

**Figura 4:** Frequência de desfechos por países

#### Habilidades e comportamentos da PBE

A maioria dos estudos apontou que, mesmo com formação prévia, os profissionais sentem necessidade de aprimorar suas habilidades em PBE. Nos Estados Unidos, cerca de 70% relataram essa necessidade, enquanto no Brasil esse número foi ainda mais expressivo (97,1%)^(1,2,22,50)^. Deficiências técnicas recorrentes incluem dificuldades na análise metodológica dos estudos, na interpretação de dados estatísticos e na utilização de estratégias eficientes de busca em bases científicas^(35)^. Tais dificuldades são mais prevalentes entre profissionais em início de carreira: na Irlanda, por exemplo, 72,8% dos terapeutas iniciantes relataram dificuldades com estatística, comparados a 42,9% dos profissionais mais experientes^(24)^.

Outro ponto relevante refere-se às fontes de informação utilizadas na prática clínica. Embora se reconheça a importância da pesquisa científica, a maioria dos profissionais ainda se baseia predominantemente na própria experiência clínica (relatada por até 99,6% dos entrevistados) e nas opiniões de colegas (até 78,7%)^(5,22,25,48)^. O uso de bases de dados científicas de alto nível, como Cochrane ou SpeechBite, ainda é limitado, especialmente no Brasil, onde plataformas como SciELO (33,6%) e Google Acadêmico (23,2%) são mais frequentemente utilizadas, refletindo tanto limitações de acesso quanto barreiras linguísticas e de familiaridade com as ferramentas^(1,52)^.

Há evidências de que a formação acadêmica influencia positivamente a adoção da PBE. A exposição à pesquisa durante a graduação, pós-graduação e residência clínica (clinical fellowship year – CFY) está associada a maior valorização e uso da PBE na prática^(2,22,25,34)^. No entanto, estudos indicam que essa exposição tende a diminuir após o período de formação inicial^(25)^. Em relação à aplicação da PBE, observou-se que ela tem sido mais incorporada na tomada de decisão terapêutica do que nas fases de avaliação, sendo frequentemente utilizada em situações de incerteza clínica ou para validar condutas previamente adotadas^(40)^.

#### Opiniões sobre a PBE

Os estudos demonstram, de maneira geral, que os fonoaudiólogos mantêm atitudes positivas em relação à PBE^(36,52)^. Profissionais de países como Malásia, Irã, Austrália e Brasil reconhecem os benefícios da prática para a qualidade do cuidado e a eficácia das decisões clínicas^(1,5,23,33)^. No Brasil, 89,3% dos entrevistados afirmaram que a PBE melhora o cuidado ao paciente, e 86,8% a consideram útil para o tratamento^(1)^. A exposição prévia à PBE durante a formação favorece atitudes mais favoráveis^(2,22)^.

Apesar da receptividade, ainda há confusão conceitual, principalmente no que se refere à integração entre evidência científica, experiência clínica e valores do paciente. Muitos profissionais continuam a associar a PBE exclusivamente à pesquisa, desconsiderando os demais componentes^(39,47)^. A percepção de sobrecarga associada ao uso da PBE também foi mencionada, sobretudo por aqueles com menor domínio do inglês ou que atuam em ambientes sem suporte institucional^(1)^.

O apoio institucional é um fator crítico. Embora alguns relatem valorização da PBE nos locais de trabalho, a percepção de que ela orienta efetivamente as decisões clínicas ainda é limitada. No Brasil, quase metade dos profissionais apontou a ausência de apoio entre colegas como uma barreira, e muitos relataram insegurança para recomendar o uso de evidências, especialmente diante de superiores hierárquicos^(1,33)^.

#### Barreiras da PBE

As barreiras à aplicação da PBE são consistentes entre os estudos e abrangem desde limitações individuais até fatores organizacionais. A mais frequente é a falta de tempo, mencionada por 91% dos fonoaudiólogos escolares nos EUA^(43)^, 71,9% na Irlanda^(24)^, 62% no Irã^(53)^ e 27,9% no Brasil, especialmente entre profissionais com menor tempo de formação^(1)^.

Dificuldades técnicas, como compreender estatísticas, avaliar a qualidade dos estudos e aplicá-los a casos individuais, foram amplamente relatadas. Até 78% dos profissionais do Reino Unido e da Irlanda relataram problemas com estatística, e nos EUA, esse índice chegou a 33%^(22,24)^. No Brasil, mais da metade dos entrevistados indicou dificuldade em aplicar resultados de pesquisa à prática clínica^(1)^.

O acesso limitado à literatura científica e a baixa familiaridade com bases especializadas também dificultam a implementação da PBE. Muitos profissionais relataram dificuldades em localizar artigos relevantes e baixa utilização de plataformas como Cochrane, PubMed e SpeechBite^(1,22,33)^. A barreira idiomática é um desafio adicional, afetando particularmente profissionais que não dominam o inglês^(1,47)^.

Entre as barreiras organizacionais, destacam-se a ausência de diretrizes institucionais formais, a falta de incentivo à prática baseada em evidências e o pouco apoio entre os colegas. Apenas 11% dos profissionais nos EUA relataram trabalhar em instituições com diretrizes específicas para PBE^(43)^. No Brasil, 48,4% citaram a ausência de apoio coletivo como obstáculo^(1)^.

Por fim, foram mencionadas limitações relacionadas à própria produção científica, como baixa replicabilidade, fragilidade metodológica e reduzida aplicabilidade prática. Essas críticas foram identificadas em estudos realizados na Irlanda, Austrália, Brasil e Coreia do Sul^(1,2,24,32,38-44,49,53)^, reforçando a importância de produzir evidências mais sólidas, relevantes e contextualizadas para a prática clínica em fonoaudiologia.

### Viés de relato

A análise do viés de publicação por meio do gráfico de funil não foi realizada, pois essa abordagem requer a condução de uma meta-análise, o que não se aplicou neste estudo. Para minimizar a possibilidade de viés de publicação, foi adotada uma estratégia de busca abrangente, incluindo sete bases de dados, literatura cinzenta e uma base em idioma distinto do inglês (LILACS).

## DISCUSSÃO

A Fonoaudiologia baseada em evidências tem sido cada vez mais discutida e disseminada nos últimos anos. Os resultados obtidos no estudo, onde a maioria dos participantes conceitua a PBE primariamente como a utilização de publicações científicas e pesquisas^(47)^, estão alinhados com uma tendência observada em diversas pesquisas. Muitos fonoaudiólogos e outros profissionais de saúde limitam a PBE à evidência de pesquisa, negligenciando a integração da experiência clínica do profissional e as preferências e valores do cliente^(40,45,47)^. Esta percepção restrita contrasta com as definições amplamente aceitas, que consideram a PBE como a integração consciente, explícita e criteriosa da melhor evidência de pesquisa externa disponível, da melhor evidência interna à prática clínica e das preferências do paciente plenamente informado. A American Speech-Language-Hearing Association (ASHA), por exemplo, enfatiza essa tríade em suas diretrizes^(6)^.

A exposição à PBE durante a graduação e, especialmente, nos primeiros anos de atuação clínica, como o período de aperfeiçoamento clínico se mostrou um fator relevante para a incorporação da PBE na rotina profissional^(1,2,22,25,34)^. Profissionais com maior formação ou treinamento direcionado em PBE tendem a relatar mais segurança na avaliação crítica de evidências e no uso dessas informações na tomada de decisões clínicas^(2,34,53)^. Aqueles com menor domínio do inglês apresentaram menos familiaridade com os princípios da PBE, evidenciando que a barreira linguística segue sendo um obstáculo importante ao acesso à literatura científica internacional de maior impacto^(1)^.

Apesar das atitudes geralmente positivas em relação à PBE, persistem obstáculos que dificultam sua implementação. A falta de tempo continua sendo uma das barreiras mais citadas, atribuída a cargas de trabalho elevadas e à pressão por produtividade^(1,2,24,38-44,49,53)^. A percepção de tempo insuficiente foi mais pronunciada entre profissionais com menos tempo de atuação, sugerindo que a experiência pode atenuar essa dificuldade. No entanto, a literatura não é unânime quanto a essa associação, com alguns trabalhos indicando que essa percepção independe do tempo de experiência^(24)^.

Outros obstáculos recorrentes incluem a escassez de estudos robustos, diretamente aplicáveis à prática clínica, dificuldade em interpretar e aplicar os achados da pesquisa^(1,2,22,24,33,38,40,44,49,53)^ e a percepção de que os artigos carecem de clareza e aplicabilidade prática. Muitos profissionais relatam que os estudos não dialogam com as realidades dos seus contextos de trabalho ou com as necessidades específicas dos pacientes^(49)^.

Além disso, a falta de apoio institucional e de uma cultura organizacional voltada para a valorização da evidência científica enfraquece a adesão à PBE. Em vários contextos, a PBE é percebida como uma exigência burocrática, sem integração efetiva com as rotinas clínicas. Soma-se a isso o acesso limitado a bases de dados, ferramentas de busca especializadas e recursos atualizados, além do custo para obtenção de materiais científicos, dificultando ainda mais a prática informada por evidências, especialmente em países de baixa e média renda^(46)^.

A barreira idiomática limita ainda mais o acesso à literatura de maior relevância internacional. Nesse cenário, a formação acadêmica desempenha papel importante: fonoaudiólogos com pós-graduação relatam menos dificuldades em adotar a PBE, reforçando que a familiaridade com pesquisa científica pode mitigar parte dessas barreiras^(1,38,47)^.

A análise do risco de viés evidenciou fragilidades metodológicas nos estudos incluídos. A maioria obteve pontuações baixas (1 a 3 em uma escala de 0 a 6), o que aponta para limitações importantes na qualidade das evidências. Apenas dois estudos atingiram 4 pontos^(24,49)^, nenhum alcançou pontuação máxima. Embora aspectos como a descrição da amostra, taxa de resposta e foco nos componentes da PBE tenham sido frequentemente atendidos, faltaram elementos fundamentais de rigor metodológico. A ausência de informações sobre não respondentes, a falta de validação dos instrumentos e a omissão do cálculo do tamanho amostral foram recorrentes, comprometendo a confiabilidade e a generalização dos achados. Essas falhas metodológicas limitam a confiabilidade das evidências disponíveis e reforçam a necessidade de estudos futuros mais rigorosos, com atenção especial ao delineamento, à validade dos instrumentos e à transparência na apresentação dos dados.

Quanto à qualidade dos estudos incluídos, observou-se uma heterogeneidade significativa. Embora alguns apresentem boa fundamentação metodológica, muitos carecem de rigor científico, seja pela ausência de delineamentos controlados^(24)^, seja pela insuficiência de detalhes que inviabilizam a replicação de intervenções. Além disso, a literatura da área frequentemente não fornece informações práticas suficientes (como materiais utilizados, protocolos de intervenção ou critérios de adaptação clínica) dificultando a implementação real das evidências^(40,49)^. Esse cenário contribui para o ceticismo de muitos clínicos quanto à aplicabilidade da pesquisa acadêmica em suas realidades.

A lacuna entre pesquisa e prática segue como um dos principais desafios da PBE. Essa desconexão é alimentada por produções acadêmicas pouco alinhadas com as necessidades da prática e por profissionais que não se sentem preparados ou apoiados para integrar a evidência científica ao seu trabalho^(39,40,44,47)^. Estratégias apontadas como promissoras incluem a produção de estudos mais aplicáveis à prática clínica real^(1,40,47)^, a descrição detalhada das intervenções^(40,49)^, a colaboração entre pesquisadores e clínicos no planejamento das investigações e a criação de recursos de fácil acesso e rápida consulta, como resumos clínicos e diretrizes atualizadas^(37,40,42,45)^.

Além disso, é fundamental promover treinamento contínuo em PBE, com foco em habilidades de busca, avaliação crítica e aplicação prática, bem como fomentar ambientes de trabalho que valorizem e sustentem o uso da evidência, por meio de grupos de discussão e mentorias^(1,40,44)^. É importante reforçar que a PBE não se limita à pesquisa publicada: ela envolve também a expertise clínica e as preferências dos pacientes. Integrar esses três pontos é fundamental para que a prática baseada em evidências seja, de fato, efetiva e sustentável.

Este estudo apresenta limitações que devem ser consideradas. A amostra, obtida por meio de questionários online e redes sociais, pode ter sofrido viés de auto-seleção, favorecendo a participação de profissionais mais familiarizados com tecnologia ou mais interessados no tema. O uso de auto-relato pode ter introduzido viés de desejabilidade social, levando os participantes a respostas mais alinhadas com o que é socialmente esperado, além da possibilidade de erros de memória ou julgamento subjetivo.

As perguntas abertas e semiestruturadas, embora úteis para explorar percepções, podem não ter capturado todas as dimensões da PBE ou explorado em profundidade as razões pelas quais determinadas barreiras foram mencionadas. A homogeneidade da amostra em termos de perfil demográfico e formação também limita a generalização dos achados para toda a população de fonoaudiólogos.

Por fim, o estudo concentrou-se nas percepções e atitudes dos participantes, não avaliando diretamente a qualidade das decisões clínicas nem distinguindo entre uso efetivo da PBE e a percepção de seu uso. Investigações futuras devem considerar abordagens metodológicas mistas, combinando questionários com observações em contexto clínico e análise de decisões terapêuticas para aprofundar a compreensão sobre o uso real da PBE na prática profissional.

Diante de tal cenário, os achados desta pesquisa reforçam que a valorização da PBE, se não acompanhada por políticas formativas e estruturais consistentes, torna-se inócua. É fundamental uma ação coordenada entre universidades, instituições de saúde e conselhos profissionais para que a formação em PBE seja integrada de forma contínua e efetiva à trajetória profissional. Paralelamente, é urgente elevar o rigor metodológico das pesquisas produzidas na área, aproximando-as das demandas da prática clínica real.

## CONCLUSÃO

Pode-se concluir então que, embora os fonoaudiólogos demonstrem atitudes geralmente positivas em relação a PBE, persistem brechas importantes no que se refere aos conhecimentos, habilidades e aplicação prática. Muitos profissionais ainda percebem a PBE de forma limitada, valorizando exclusivamente a evidência científica, sem integrar de forma adequada a experiência clínica e as preferências dos pacientes, elementos centrais ao conceito da PBE.

As principais barreiras identificadas foram falta de tempo, baixa familiaridade com métodos de busca e avaliação crítica, domínio insuficiente do inglês, e carência de apoio institucional são pontos que se repetem em diferentes contextos e impactam diretamente na implementação eficiente da PBE. Além disso, observou-se uma heterogeneidade metodológica nos estudos incluídos, com pontuações baixas no risco de viés e escassez de validação dos instrumentos utilizados, o que reduz a confiança geral na evidência disponível.

Apesar disso, a exposição prévia à PBE durante a formação acadêmica se mostrou um fator importante e promissor para melhores atitudes e comportamentos relacionados a prática, sugerindo que o investimento em capacitação específica pode mitigar parte das barreiras enfrentadas e trazer mais confiança aos profissionais interessados.

## Apêndice 1 Estratégias de busca nas bases de dados e literatura cinzenta

**Table:**

**Bases de dados**	**Busca** (19 de novembro de 2024)
**Embase**	#1. 'evidence based'/exp OR 'evidence based' OR 'evidence-based practice'/exp OR 'evidence-based practice' OR 'evidence based practice'/exp OR 'evidence based practice' OR 'evidence based medical practice'/exp OR 'evidence based medical practice' OR 'evidence-based medicine'/exp OR 'evidence-based medicine' OR 'evidence based medicine'/exp OR 'evidence based medicine'
	#2. 'attitude' OR 'attitudes' OR 'sentiment' OR 'sentiments' OR 'opinions' OR 'opinion' OR 'behavior' OR 'self-efficacy' OR 'knowledge' OR 'perception' OR 'research' OR 'barrier' OR 'skill' OR 'intention' OR 'belief' OR 'self-reported practice' OR 'education' OR 'interest' OR 'resources' OR 'participation' OR 'abilit' OR 'implementation' OR 'implementing' OR 'access' OR 'problem'
	#3. 'speech therapy'/exp OR 'logopedic education' OR 'logopedic training' OR 'speech education' OR 'speech training' OR 'therapy, speech' OR 'speech therapy' OR 'speech, language and hearing sciences' OR 'speech-language pathology' OR 'speech language pathology' OR 'language pathology' OR 'speech pathology'
	#4. #1 AND #2 AND #3
	#5. #4 AND [embase]/lim NOT ([embase]/lim AND [medline]/lim)
**LILACS**	#1.MH:"Prática Clínica Baseada em Evidências" OR "Prática Clínica Baseada em Evidências" OR "Assistência Sanitária Baseada em Evidência" OR "Assistência Sanitária Baseada em Evidências" OR "Atenção à Saúde Baseada em Evidências" OR "Atenção à Saúde Baseada na Evidência" OR "Cuidado à Saúde Baseado em Evidências" OR "Cuidados de Saúde Baseados em Evidências" OR "Cuidados de Saúde Baseados na Evidência" OR "Prática Médica Baseada em Evidências" OR "Saúde Pública Baseada em Evidência" OR "Saúde Pública Baseada em Evidências" OR "Saúde Pública Baseada na Evidência" OR "Evidence-Based Practice" OR "Práctica Clínica Basada en la Evidencia" OR MH:H02.249$
	#2. MH:Atitude OR Atitude OR Atitudes OR Opinião OR Opiniões OR "Sentimento" OR "Actitud" OR "Opiniones" OR "Sentimiento" OR "Attitude" OR "Attitudes" OR "Opinion" OR "Opinions" OR "Sentiment" OR "Sentiment" OR "Sentiments" OR "behavior" OR "self-efficacy" OR "self-efficacy" OR "knowledge" OR "perception" OR "research" OR "barrier" OR "skill" OR "intention" OR "belief" OR "self-reported practice" OR "education" OR "interest" OR "resources" OR OR "participation" OR "abilit" OR "implementation" OR "implementing" OR "access" OR "problem" OR MH:F01.100$
	#3.MH:Fonoaudiologia OR Fonoaudiologia OR "Speech, Language and Hearing Sciences" OR Fonoaudiología OR "Ciência da Fala e Audição" OR "Ciência da Fonação e Audição" OR "Estudo da Fala e Audição" OR "Estudo da Fala e da Audição" OR "Estudo da Fonação e Audição" OR "Estudos da Fala e Audição" OR "Estudos da Fala e da Audição" OR "Estudos da Fonação e Audição" OR "Estudos da Fonação e da Audição" OR "Patologia da Fala e Linguagem e Audiologia" OR MH:SH1.020.020.040.045$ OR MH:"Patologia da Fala e Linguagem" OR "Speech-Language Pathology" OR "Patología del Habla y Lenguaje" OR Patologia da Fala" OR "Patologia da Linguagem" OR MH:H02.010.750$
	#4. #1 AND #2 AND #3
**LIVIVO**	("Evidence-Based Practice" OR "Evidence Based Practice" OR "Evidence-Based Health Care" OR "Evidence Based Health Care" OR "Evidence-Based Healthcare" OR "Evidence Based Healthcare" OR "Evidence Based Health Care Management" OR "Evidence Based Healthcare Management" OR "Evidence-Based Medicine" OR "Evidence Based Medicine") AND ("Attitude" OR "Attitudes" OR "Sentiment" OR "Sentiments" OR "Opinions" OR "Opinion" OR "behavior" OR "self-efficacy" OR "self-efficacy" OR "knowledge" OR "perception" OR "research" OR "barrier" OR "skill" OR "intention" OR "belief" OR "self-reported practice" OR "education" OR "interest" OR "resources" OR "participation" OR "abilit" OR "implementation" OR "implementing" OR "access" OR "problem") AND ("Speech Therapy" OR "Speech Therapies" OR "Speech, Language and Hearing Sciences" OR "Speech-Language Pathology" OR "Speech Language Pathology" OR "Language Pathology" OR "Speech Pathology")
**PubMed/ Medline**	#1. "Evidence-Based Practice"[Mesh] OR "Evidence Based Practice" OR "Evidence-Based Health Care" OR "Evidence Based Health Care" OR "Evidence-Based Healthcare" OR "Evidence Based Healthcare" OR "Evidence Based Health Care Management" OR "Evidence Based Healthcare Management" OR "Evidence-Based Medicine"[Mesh] OR "Evidence Based Medicine"
	#2. "Attitude"[Mesh] OR "Attitudes" OR "Sentiment" OR "Sentiments" OR "Opinions" OR "Opinion" OR "behavior" OR "self-efficacy" OR "self-efficacy" OR "knowledge" OR "perception" OR "research" OR "barrier" OR "skill" OR "intention" OR "belief" OR "self-reported practice" OR "education" OR "interest" OR "resources" OR "participation" OR "abilit" OR "implementation" OR "implementing" OR "access" OR "problem"
	#3. "Speech Therapy"[Mesh] OR "Speech Therapies" OR "Speech, Language and Hearing Sciences" OR "Speech-Language Pathology"[Mesh] OR "Speech Language Pathology" OR "Language Pathology" OR "Speech Pathology"
	#4. #1 AND #2 AND #3
**SciELO**	"Evidence-Based Practice" OR "Evidence Based Practice" OR "Evidence-Based Health Care" OR "Evidence Based Health Care" OR "Evidence-Based Healthcare" OR "Evidence Based Healthcare" OR "Evidence Based Health Care Management" OR "Evidence Based Healthcare Management" OR "Evidence-Based Medicine" OR "Evidence Based Medicine" (Topic) AND "Attitude" OR "Attitudes" OR "Sentiment" OR "Sentiments" OR "Opinions" OR "Opinion" OR "behavior" OR "self-efficacy" OR "self-efficacy" OR "knowledge" OR "perception" OR "research" OR "barrier" OR "skill" OR "intention" OR "belief" OR "self-reported practice" OR "education" OR "interest" OR "resources" OR "participation" OR "abilit" OR "implementation" OR "implementing" OR "access" OR "problem" (Topic) AND "Speech Therapy" OR "Speech Therapies" OR "Speech, Language and Hearing Sciences" OR "Speech-Language Pathology" OR "Speech Language Pathology" OR "Language Pathology" OR "Speech Pathology" (Topic)
**Scopus**	(TITLE-ABS-KEY (( "Evidence-Based Practice" OR "Evidence Based Practice" OR "Evidence-Based Health Care" OR "Evidence Based Health Care" OR "Evidence-Based Healthcare" OR "Evidence Based Healthcare" OR "Evidence Based Health Care Management" OR "Evidence Based Healthcare Management" OR "Evidence-Based Dentistry" OR "Evidence Based Dentistry" OR "Evidence-Based Medicine" OR "Evidence Based Medicine" )) AND TITLE-ABS-KEY (( "Attitude" OR "Attitudes" OR "Sentiment" OR "Sentiments" OR "Opinions" OR "Opinion" OR "behavior" OR "self-efficacy" OR "self-efficacy" OR "knowledge" OR "perception" OR "research" OR "barrier" OR "skill" OR "intention" OR "belief" OR "self-reported practice" OR "education" OR "interest" OR "resources" OR "participation" OR "abilit" OR "implementation" OR "implementing" OR "access" OR "problem" )) AND TITLE-ABS-KEY (( "Dentists" OR "dentist" )))
**Web of Science**	"Evidence-Based Practice" OR "Evidence Based Practice" OR "Evidence-Based Health Care" OR "Evidence Based Health Care" OR "Evidence-Based Healthcare" OR "Evidence Based Healthcare" OR "Evidence Based Health Care Management" OR "Evidence Based Healthcare Management" OR "Evidence-Based Medicine" OR "Evidence Based Medicine" (Topic) and "Attitude" OR "Attitudes" OR "Sentiment" OR "Sentiments" OR "Opinions" OR "Opinion" OR "behavior" OR "self-efficacy" OR "self-efficacy" OR "knowledge" OR "perception" OR "research" OR "barrier" OR "skill" OR "intention" OR "belief" OR "self-reported practice" OR "education" OR "interest" OR "resources" OR "participation" OR "ability" OR "implementation" OR "implementing" OR "access" OR "problem" (Topic) and "Speech Therapy" OR "Speech Therapies" OR "Speech, Language and Hearing Sciences" OR "Speech-Language Pathology" OR "Speech Language Pathology" OR "Language Pathology" OR "Speech Pathology" (Topic) |
**Google Scholar**	"Evidence-Based Practice" AND "Attitude" OR "Sentiment" OR "Opinions" OR "behavior" AND "Speech Therapy" OR "Speech-Language Pathology"
**ProQuest**	"Evidence-Based Practice" OR "Evidence Based Practice" OR "Evidence-Based Health Care" OR "Evidence Based Health Care" OR "Evidence-Based Healthcare" OR "Evidence Based Healthcare" OR "Evidence Based Health Care Management" OR "Evidence Based Healthcare Management" OR "Evidence-Based Medicine" OR "Evidence Based Medicine" (Topic) AND "Attitude" OR "Attitudes" OR "Sentiment" OR "Sentiments" OR "Opinions" OR "Opinion" OR "behavior" OR "self-efficacy" OR "self-efficacy" OR "knowledge" OR "perception" OR "research" OR "barrier" OR "skill" OR "intention" OR "belief" OR "self-reported practice" OR "education" OR "interest" OR "resources" OR "participation" OR "abilit" OR "implementation" OR "implementing" OR "access" OR "problem" (Topic) AND "Speech Therapy" OR "Speech Therapies" OR "Speech, Language and Hearing Sciences" OR "Speech-Language Pathology" OR "Speech Language Pathology" OR "Language Pathology" OR "Speech Pathology" (Topic)

## Apêndice 2 Artigos excluídos e motivo da exclusão (n = 22)

**Table:**

**Autor, Ano**	**Razão da exclusão**
Abrams et al., 2005^1^	3
Alary Gauvreau et al., 2019^2^	3
Allison, 2021^3^	3
Baker et al., 2011^4^	3
Beushausen, 2005^5^	3
Beushausen, 2014^6^	3
Boisvert et al., 2017^7^	3
Borgelt et al., 2015^8^	3
Borgetto et al., 2016^9^	2
Brackenbury et al., 2008^10^	3
Brock, 2025^11^	2
Campbell et al., 2017^12^	2
Georgieva et al., 2011^13^	3
Gomez et al., 2011^14^	2
Guo et al., 2008^15^	2
Harding et al., 2014^16^	1
Kwok et al., 2022^17^	2
Lof, 2011^18^	2
Meline, 2003^19^	2
Mullen, 2005^20^	2
Ratner, 2005^21^	2
Trembath et al., 2016^22^	2

**Legenda:** 1. Não envolvam estudantes ou profissionais da fonoaudiologia; 2. Não relatem conhecimentos, habilidades, comportamento, opiniões e barreiras em fonoaudiologia em relação à PBE; 3. Sejam revisões, cartas ao editor, relatos de casos, séries de casos, opiniões de especialistas e diretrizes.

**REFERÊNCIAS DO** **APÊNDICE 2**

1. Abrams HB, McArdle R, Chisolm TH. From outcomes to evidence: establishing best practices for audiologists. Semin Hear. 2005;26(4):207–13.

2. Alary Gauvreau C, le Dorze G, Kairy D, Croteau C. Evaluation of a community of practice for speech-language pathologists in aphasia rehabilitation: a logic analysis. BMC Health Serv Res. 2019;19(1):530.

3. Allison LHZ. Evidence-Based Practice for Speech-Language Pathologists: A Survey of Access and Implementation [dissertation]. Auburn: Auburn University; 2021.

4. Baker E, McLeod S. Evidence-based practice for children with speech sound disorders: Part 2. Application to clinical practice. Lang Speech Hear Serv Sch. 2011;42(2):140–51.

5. Beushausen U. “Evidence-based practice” in speech pathology – myth and reality. Forum Logopädie. 2005;19(2):6-11.

6. Beushausen U. Evidence-based speech-language pathology: prospects and risks. L.O.G.O.S. Interdisziplinär. 2014;22(2):96-104.

7. Boisvert I, Clemesha J, Lundmark E, Crome E, Barr C, McMahon CM. Decision-making in audiology: balancing evidence-based practice and patient-centered care. Trends Hear. 2017;21:1–14.

8. Borgelt T. When there is no evidence available: evidence-based practice scenarios in day-to-day speech therapy care. Forum Logopädie. 2015;29(1):24–29.

9. Borgetto B, Spitzer L, Pfingsten A. The research pyramid: how to make evidence useful for the practice of speech and language therapy. Forum Logopädie. 2016;30(1):24-28.

10. Brackenbury T, Burroughs E, Hewitt LE. A qualitative examination of current guidelines for evidence-based practice in child language intervention. Lang Speech Hear Serv Sch. 2008;39(1):96–109.

11. Brock AS. Using case-based learning to teach evidence-based practice: a pilot study. Clin Teach. 2025;22(1):e13842.

12. Campbell WN, Douglas NF. Supporting evidence-based practice in speech-language pathology: a review of implementation strategies for promoting health professional behavior change. Evid Based Commun Assess Interv. 2017;11(3–4):43–51.

13. Georgieva D, Stefanovska A. Evidence-based assessment of voice disorders: a theoretical overview and model. J Spec Educ Rehabil. 2011;12(1–2):32-39.

14. Gomez M, McCabe P, Purcell A. A survey of the clinical management of childhood apraxia of speech in the United States and Canada. J Commun Disord. 2022;96:106181.

15. Guo R, Bain BA, Willer J. Results of an assessment of information needs among speech-language pathologists and audiologists in Idaho. J Med Libr Assoc. 2008;96(2):138–144.

16. Harding KE, Porter J, Horne-Thompson A, Donley E, Taylor NF. Not enough time or a low priority? Barriers to evidence-based practice for allied health clinicians. J Contin Educ Health Prof. 2014;34(4):224–31.

17. Kwok EY, Moodie ST, Cunningham BJ, Oram Cardy J. Barriers and facilitators to implementation of a preschool outcome measure: an interview study with speech-language pathologists. J Commun Disord. 2022;95:106160.

18. Lof GL. Science-based practice and the speech-language pathologist. Int J Speech Lang Pathol. 2011;13(3):189–96.

19. Meline T, Paradiso T. Evidence-based practice in schools. Lang Speech Hear Serv Sch. 2003;34(4):273–83.

20. Mullen R. Survey tests members’ understanding of evidence-based practice: a systematic approach to earmold selection. Leader. 2005;10(15):4-14.

21. Ratner NB. Evidence-based practice in stuttering: some questions to consider. J Fluency Disord. 2005;30(3):163–88.

22. Trembath D, Hawtree R, Arciuli J, Caithness T. What do speech-language pathologists think parents expect when treating their children with autism spectrum disorder? Int J Speech Lang Pathol. 2016;18(3):250-59.

## Apêndice 3 Descrição das características relacionadas ao conhecimento, habilidades, atitudes, opiniões e barreiras relatadas por cada estudo (n=31)

**Table:**

**Nome, Data, País**	**Conhecimentos e habilidades**	**Comportamentos**	**Opiniões**	**Barreiras**
Alaidary, Abdulsalam et al.^(2)^, 2020, Arabia Saudita	93.8% completou um curso de metodologia de pesquisa;	As principais fontes de informação para decisões clínicas foram: experiência clínica pessoal, diretrizes de prática clínica, recursos da Internet, livros didáticos e estudos de pesquisa;	90% concordaram que é importante reservar tempo para a PBE e que sua aplicação deve ser incentivada;	60% relataram não ter tempo para ler literatura de pesquisa no trabalho;
	87.5% foi exposta a artigos;		60.4% consideram os achados de pesquisa relevantes para a prática diária;	52.1% tinham ferramentas para pesquisa online no trabalho;
		O treinamento específico em PBE aumentou o uso de estudos de pesquisa para guiar decisões clínicas.	41.7% acreditam que a PBE aumentará sua carga de trabalho.	A aplicação de achados de pesquisa na prática é percebida como desafiadora, com 37.5% concordando e 33.3% sendo neutros.
	79.2% se formaram com entendimento e conhecimento de PBE;			
	66.7% tiveram treinamento em PBE			
	66.7% sente-se confortável em ler artigos.			
Bennett et al.^(31)^, 2019, Austrália	Reconhecem a importância de ferramentas padronizadas e avaliações objetivas;	Devido às pressões de tempo, há um alto uso de ferramentas de triagem em vez de avaliações completas;	Eles consideram que o "melhor cuidado" (baseado em pesquisa) deve ser equilibrado com o "cuidado responsável", especialmente para idosos frágeis com problemas cognitivos;	O acesso a, e a dinâmica de, uma equipe multiprofissional eficaz e uma rede de apoio profissional;
		Educar equipe, familiares e cuidadores é essencial na PBE, especialmente em cuidados comunitários e residenciais com manejo limitado.		
			O estudo propõe um modelo ampliado de decisão baseada em evidências para idosos, considerando contexto clínico e atuação da equipe.	A falta de tempo e recursos limitados;
				A falta de validação de ferramentas de avaliação e terapia para populações idosas complexas é uma barreira;
	Questiona-se a utilidade clínica de ferramentas feitas para adultos, pois idosos têm comorbidades e necessidades complexas.			
				Falta de recursos, localização e baixa prioridade limitam a educação continuada em fonoaudiologia para idosos, agravada por custos e acessibilidade.
Chan A. K. et al.^(32)^, 2013, Austrália	A maioria acredita possuir as habilidades fundamentais para encontrar e avaliar a literatura;	Na falta de evidências de alta qualidade, 98% recorrem à própria experiência clínica;	96% dos fonoaudiólogos às vezes ou sempre usaram a abordagem de terapia que acreditavam ser a mais eficaz;	88% acreditavam na falta de evidência de alta qualidade;
				86% relataram que a evidência nem sempre está acessível;
		Outras fontes usadas sem evidência de alta qualidade são: consultar livros didáticos 91%, colegas 89% e participar de eventos de desenvolvimento profissional 83%.	As escolhas de manejo são influenciadas pela experiência clínica do profissional, razões centradas no paciente e a presença de evidências externas.	
				81% informam falta de tempo para ler a literatura;
				A falta de financiamento para treinamento formal em programas também foi mencionada.
	A falta de treinamento e o acesso limitado a workshops especializados impedem a oferta do tratamento “ideal”.			
Cheung et al.^(33)^, 2013, Austrália	97% concorda que a aplicação da PBE é necessária;	23% acreditam que a maioria dos fonoaudiólogos aplica a PBE no atendimento a crianças com TEA;	A PBE é vista como fundamental para uma prática eficaz e ética;	Falta de compreensão por parte da gestão;
	64% receberam treinamento em PBE;	10% acessam recursos de PBE raramente; 46% acessam semanalmente;	97% concordam que a PBE é necessária na prática de fonoaudiologia;	44% não sentem que têm tempo para revisar a literatura antes de tomar decisões clínicas;
	48% tinham treinamento adequado para usar ferramentas eletrônicas de busca de pesquisa eficientemente;	Profissionais dependem do julgamento e experiência pela falta de pesquisa qualificada em autismo.	41% percebem uma divisão entre pesquisa e prática;	18% disseram que gerenciar a lista de espera é prioridade sobre intervenções baseadas em evidências.
	76% busca evidências de pesquisa regularmente		82% indicaram que a PBE é apoiada em seus locais de trabalho;	28% indicaram que lhes faltavam as ferramentas eletrônicas necessárias para encontrar pesquisa apropriada;
	Fonoaudiólogos com mais de 10 anos de experiência sentem-se mais confiantes ao recomendar o uso da PBE a colegas, especialmente a juniores.		66% indicaram que as decisões de serviço são baseadas em evidências de pesquisa;	34% indicaram que seus locais de trabalho não tinham financiamento suficiente para atividades de PBE,
			Existe uma crítica comum de que gerentes e administradores não compreendem a importância da PBE.	
Choi et al.^(34)^, 2015, Coréia do Sul	Atitude positiva à PBE está ligada à exposição à pesquisa e prática durante graduação e estágio;	A experiência clínica guia decisões mais que estudos de pesquisa;	Foram relatadas atitudes geralmente positivas em relação à PBE;	Os participantes não relataram altas barreiras para o uso da PBE;
	A percepção de falta de conhecimento e habilidades para a PBE (como busca e avaliação de literatura) não foi considerada uma barreira significativa;	Mais de 50% usam com frequência experiência clínica e livros didáticos como principais fontes.	A afirmação com maior concordância foi que "manter-se atualizado com as tendências de pesquisa em patologia da fala-linguagem é uma responsabilidade profissional ao longo da vida";	A falta de quantidade e qualidade dos estudos na área de interesse foi percebida como uma barreira importante;
		Menos de 10% usam pesquisas com frequência, sendo a fonte menos consultada;	Eles tenderam a discordar que "os resultados de pesquisa não estão relacionados à minha prática clínica e profissionalismo".	Quem usava menos bases de dados ou tinha pouca experiência em PBE percebia mais barreiras;
		Fonoaudiólogos com maior tempo de experiência clínica apresentaram um uso *menor* de PBE.		Fonoaudiólogos que participaram de conferências nos últimos 2 anos relataram *maiores* barreiras do que aqueles que não participaram.
Chu et al.^(23)^, 2021, Japão e Malásia	Fonoaudiólogos com Mestrado ou Doutorado tendem a ter mais conhecimento sobre PBE;	A maioria dos fonoaudiólogos na Malásia e no Japão baseia suas decisões clínicas principalmente na experiência pessoal e em sugestões de colegas, em vez de pesquisas publicadas;	Fonoaudiólogos da Malásia demonstraram atitudes significativamente mais positivas em relação à PBE do que os do Japão (p < 0,01);	Em ambos os países, fonoaudiólogos com maior nível educacional tendem a perceber menos barreiras à PBE;
	Sugere-se que o treinamento, especialmente em nível de Mestrado, pode aumentar a auto eficácia e as habilidades em PBE;	Embora não significativo, mais fonoaudiólogos malaios relataram aprender sobre PBE por meio da internet e workshops do que os japoneses.	Fonoaudiólogos de ambos os países têm atitudes positivas em relação à PBE, reconhecendo seu potencial para melhorar os resultados clínicos e a relevância das pesquisas para a prática;	Apenas 27% dos fonoaudiólogos malaios e 34% dos japoneses relataram falta de tempo para praticar a PBE, indicando que o tempo não é uma barreira significativa para eles, diferentemente de estudos nos EUA;
	Habilidades essenciais para a PBE incluem: encontrar informações válidas, interpretar estatísticas e aplicar informações clinicamente;		Mulheres malaias que trabalham em período integral e em ambientes governamentais relataram maior motivação para desenvolver habilidades em PBE;	Outras barreiras à implementação da PBE incluem dificuldade em aplicar pesquisas na prática, evidências limitadas, restrições no trabalho, acesso restrito a recursos, cultura organizacional desfavorável e falta de conhecimento e habilidades.
	Programas de treinamento recentes na Malásia estão reforçando a importância das habilidades em PBE.		Fonoaudiólogos malaios com Mestrado demonstraram percepção mais positiva sobre a PBE do que os com graduação (p < 0,05).	
Chu et al.^(35)^, 2022, Malásia e EUA	Estudantes dos EUA relataram maior conhecimento sobre PBE em comparação aos da Malásia.	A evidência de pesquisa não tem sido a principal influência na tomada de decisão clínica;	Fonoaudiólogos nos EUA mostraram interesse por mais treinamento e recursos para apoiar a PBE;	Dificuldade em transferir as descobertas de pesquisa para a prática clínica;
	Fonoaudiólogos devem conhecer o processo de pesquisa e aplicá-lo na prática clínica.	50% dos fonoaudiólogos em casos de traqueostomia usam evidências científicas;	A maioria dos estudantes nos EUA considerou a aplicação da PBE necessária e que ela contribui para melhores resultados para os clientes;	Ter evidências limitadas, conflitantes ou irrelevantes;
	As principais habilidades citadas são: formular questões clínicas, buscar evidências relevantes, avaliar criticamente as evidências, aplicar as evidências e avaliar o resultado do processo;	A decisão clínica tende a se basear mais na experiência pessoal e em métodos aprendidos na universidade do que em evidências científicas;	Estudantes da Malásia e dos EUA mostraram dúvidas sobre a validade de basear a prática em protocolos utilizados por outros profissionais ao longo do tempo;	A restrição de tempo é relatada como uma barreira;
	A atividade em sala de aula foi eficaz para melhorar a compreensão dos estudantes sobre a PBE, promovendo também a aprendizagem colaborativa entre colegas.	99,6% dos fonoaudiólogos consideram a experiência de colegas uma fonte clínica primária e útil de informação;	Ambos os grupos, da Malásia e dos EUA, ficaram satisfeitos com o uso da PBE na prática clínica e demonstraram interesse e motivação pela atividade.	Restrições no local de trabalho e acesso limitado a recursos são outras barreiras.
		Estudantes pretendem aplicar, no futuro, as habilidades adquiridas para utilizar a PBE na prática clínica.		
Cormack, Ailbhe^(36)^. 2010, Texas, EUA	Demonstrou que clínicos que completam mais cursos em PBE e metodologia de pesquisa são mais propensos a implementar a PBE.	Clínicos que se envolvem em mais atividades de pesquisa e recebem mais apoio em seu local de trabalho são mais propensos a implementar a PBE;	A maioria dos fonoaudiólogos expressou valores positivos em relação à PBE, reconhecendo sua importância para uma prática clínica eficaz e atualizada;	Relataram não ter tempo suficiente durante o expediente para ler e aplicar literatura científica em seus casos clínicos;
		A ASHA mandatou os fonoaudiólogos a incorporar a PBE em sua tomada de decisão clínica.	Muitos participantes acreditam que a PBE melhora a qualidade do atendimento ao paciente e deve ser integrada à prática diária.	Muitos profissionais não têm acesso fácil a bancos de dados científicos, revistas especializadas ou bibliotecas;
				Aqueles com menos formação em metodologia de pesquisa e estatística demonstraram menor probabilidade de usar a PBE;
				Relataram dificuldade em interpretar e aplicar os resultados de pesquisas científicas na prática clínica.
Cunningham, B^(37)^, 2019, Canadá	89% diz que a maioria sentia que tinha as habilidades necessárias para implementar;	Após o módulo de aprendizado online, os fonoaudiólogos relataram fortes intenções de implementar os procedimentos;	91% disseram que os procedimentos refletem uma abordagem clínica eficaz;	Para o Monitoramento de Resultados: Poucas barreiras; maior parte das respostas foi positiva.
	90% relatou que regularmente usava ferramentas padronizadas.	O estudo destacou a importância de envolver clínicos na criação de avaliações baseadas em evidências para identificar barreiras antes da implementação em larga escala. As percepções analisadas são prévias à implementação, que será avaliada após um ano de testes.	87% acham que o processo beneficia famílias e crianças atendidas.	Para o Teste de Vulnerabilidade Individual: Barreiras em todos os domínios: ambiente, inovação e habilidades. Exemplos: Tempo necessário para aplicar e interpretar os testes. Falta de materiais ou apoio administrativo. Percepção de que os testes são difíceis de integrar à rotina.
Durieux et al.^(38)^, 2015, Bélgica	88,2% dos fonoaudiólogos belgas relatou nunca ter ouvido falar da PBE anteriormente;	96,9% dos fonoaudiólogos buscam soluções para seus problemas na prática;	Entre os 11,8% que já haviam ouvido falar de PBE, 16,3% a consideravam essencial, 24,5% interessante, 22,5% interessante, mas inviável, 36,7% não tinham conhecimento suficiente para opinar e nenhum a considerou sem interesse;	Falta de tempo (54,2%);
	Em uma escala de 1 a 10 os fonoaudiólogos atribuíram média de 6,9 para a habilidade de buscar informação científica, 6,7 para avaliá-la e 7,3 para aplicá-la na prática;	Para suprir necessidades de informação, a maioria recorre a recursos próprios: experiência pessoal (82.2%), biblioteca pessoal (72.4%), e colegas no local de trabalho (78.0%);	Os fonoaudiólogos geralmente estão satisfeitos com suas estratégias de busca de informação;	Desconhecimento dos recursos da área (43,9%);
	Maior nível educacional e mais tempo em formação continuada estão associados a maior familiaridade com a PBE.	Na internet, os profissionais preferem usar mecanismos de busca gerais: 48,8% para artigos científicos e 43,2% para outros documentos, enquanto apenas 5,04% utilizam bases de dados bibliográficas especializadas.	Há um interesse expresso por treinamentos em PBE (73.0%), busca de informações (65.2%) e leitura crítica de informações científicas (49.9%).	Baixa proficiência em inglês (42,4%);
				Dificuldade de acesso a ferramentas de busca especializadas (38,3%);
				Custo da informação (37,3%);
				Dificuldade em selecionar documentos relevantes (35,9%);
				Dificuldade em avaliar a qualidade científica da informação (32,5%) e falta de habilidades no uso de ferramentas de busca especializadas (27,0%);
				Falta de conhecimento ou competência (29,5%).
Foster, A^(39)^, 2015, Austrália	Os fonoaudiólogos apresentaram uma visão limitada da PBE, centrada na pesquisa científica e negligenciando a experiência clínica e as preferências dos pacientes;	Há uma lacuna entre a evidência e a prática na gestão da afasia aguda, com os serviços destoando das recomendações de melhores práticas;	Expressam o desejo de praticar a PBE, considerando-a um “objetivo aspiracional” e uma “melhor prática que deveríamos estar seguindo”;	O desempoderamento é o tema central, surgindo da visão limitada da PBE, da relação difícil com a pesquisa e da percepção de incapacidade para promover mudanças;
	Percepção de falta de habilidades para buscar pesquisas e dificuldade em compreender as recomendações da literatura científica.	A relação com a pesquisa é descrita como tensa, pois muitos percebem que os estudos não se aplicam diretamente à prática clínica;	Dão grande valor e consideram importante a pesquisa e a PBE;	Falta de recursos, equipe e tempo;
		Demonstram preferência por fontes de pesquisa compiladas e pré-avaliadas, como diretrizes e revisões sistemáticas, em vez da literatura original.	Há um sentimento de mal-estar profissional entre os fonoaudiólogos, devido à percepção de que não conseguem implementar as recomendações baseadas em evidências;	Prioridade organizacional para outras condições como disfagia;
			A pesquisa é vista por muitos como pouco útil na prática clínica e distante da realidade profissional. Ainda assim, reconhece-se um imperativo ético em oferecer cuidados baseados em evidências.	Escassez percebida de literatura relevante sobre afasia aguda;
				Dificuldades em buscar, avaliar e aplicar recomendações;
				Atitudes que desestimulam a busca por literatura;
				Falta de supervisão e mentoria.
Fulcher-Rood et al.^(40)^, 2020, EUA	64% definiu a PBE como o uso de estratégias comprovadamente eficazes, com ênfase nos achados de pesquisa;	Usam a PBE para decisões de tratamento, mas não para avaliação;	72% considerou a pesquisa como o alicerce do modelo de PBE;	A falta de tempo foi a barreira mais citada por 64%, causada por alta carga de trabalho, papelada e outras demandas, levando muitos a realizar atividades de PBE fora do horário de trabalho;
	8% incluiu todas as três fontes de evidência em sua definição de PBE;	Leem dois artigos de pesquisa por mês;	48% consideraram a pesquisa valiosa principalmente para apoiar suas decisões clínicas diárias;	16% relatam falta de financiamento;
	Relataram dificuldade em traduzir os achados de pesquisa para a prática;	Entre 25 participantes, as fontes de busca de evidência foram: buscas gerais (ex: Google) 56%, site da ASHA 52%, periódicos revisados por pares 44% e sites relacionados à fonoaudiologia 32%.	A pesquisa foi considerada menos valiosa quando não atendia às necessidades clínicas atuais (28%) ou quando parecia inviável para o ambiente de trabalho, especialmente no contexto escolar (28%).	16% citam incapacidade de replicar os métodos de estudos de pesquisa;
	36% implementam a pesquisa principalmente por falta de conhecimento específico.	Motivos para não implementar a pesquisa foram a incompatibilidade das recomendações com as necessidades dos clientes (32%) e a dificuldade de aplicar estratégias no ambiente escolar por limitações práticas (28%).		Dificuldade em traduzir os achados de pesquisa para a prática clínica.
Gomez et al.^(41)^, 2019, Austrália e Nova Zelândia	A maioria considerou que sua formação os preparou apenas parcialmente (58%) ou nada (35%) para tratar CAS, e apenas 7% sentiram-se devidamente preparados;	71% buscaram informações de colegas;	Muitos valorizam a evidência de pesquisa empírica;	49% relatam estar "muito ocupado" para pesquisar/ler a literatura sobre CAS;
	48% relataram dificuldade em interpretar análises estatísticas;	68% usa sua própria experiência clínica;	35% consideraram níveis mais baixos de evidência como adequados, bem como níveis mais altos;	Dificuldade em "interpretar análises estatísticas": 48%.
	Há uma responsabilidade das instituições acadêmicas em preparar os estudantes para acessar e interpretar a literatura de forma eficaz.	62% utilizam artigos de periódicos e 52% workshops presenciais;	21% estavam inseguros sobre como julgar a adequação da pesquisa empírica.	46% apresentam dificuldade em "acessar artigos/relatórios" para tratamento de CAS facilmente;
		62% participaram de atividades de desenvolvimento profissional em CAS.		Percepção de evidência de alta qualidade limitada para o tratamento de CAS (38%);
				Desafio de compreender a pesquisa relatada para CAS (35%).
Greenwell, T. et al.^(22)^, 2021, EUA	80% indicaram que entendiam o que constitui a PBE;	A exposição à PBE na pós-graduação e o treinamento ao longo da carreira foram fatores que previram significativamente seu uso na prática;	89.3% via a PBE favoravelmente, indicando que eram "advogados da PBE";	54% inclui a insuficiência de tempo para pesquisar e ler a literatura foi a barreira mais citada;
	81% estavam confiantes em determinar a intervenção ótima diante de evidências conflitantes.	As três fontes mais utilizadas na PBE foram as preferências do cliente (média 4,43), a evidência externa/pesquisa (média 3,99) e a experiência clínica (média 3,87);	80% dos respondentes indicaram que compreendiam o que constitui a PBE;	43% cita o tamanho da carga de trabalho foi a segunda barreira mais citada;
		65.4% receberam treinamento em PBE como parte de uma aula na pós-graduação.	81% expressaram confiança em sua capacidade de determinar a intervenção ideal diante de evidências conflitantes.	35.37% citaram o acesso a artigos de periódicos no trabalho;
				15.56% identificaram a falta de treinamento como uma barreira.
Hegarty, N. et al.^(42)^, 2020, Reino Unido	Relataram dificuldade em "entender" abordagens mais complexas;	Há uma tendência a manter abordagens tradicionais, enquanto abordagens mais novas ou complexas são frequentemente negligenciadas;	Perceberam a qualidade da literatura existente como "não robusta";	A insuficiência de tempo para pesquisar e ler a literatura;
	Sugeriu-se que os fonoaudiólogos desenvolvam suas próprias bases de evidência (ex: estudos de caso único) e aprimorem suas habilidades para coproduzir pesquisas clinicamente viáveis.	As decisões clínicas são baseadas nas próprias experiências e nas de seus colegas.	Combinar pesquisa, experiência clínica e preferências da criança/pais pode resultar em intervenções mais eficazes;	As restrições relacionadas ao serviço, como recursos limitados, grandes cargas de trabalho e a dificuldade em replicar a pesquisa na prática real;
			Consideraram que um recurso online e baseado em evidências para apoiar a tomada de decisões clínicas seria útil.	Dificuldades de acesso a materiais de intervenção desempenham um papel na escolha da intervenção;
				Níveis de confiança (em abordagens desconhecidas ou na replicação clínica);
				Dificuldades em pesquisar a literatura e em se manter atualizado com as pesquisas atuais.
Hoffman, L. M. et al.^(43)^, 2013, EUA	Profissionais em início de carreira (62%) relataram fazer mais cursos de pós-graduação relacionados à PBE do que profissionais experientes (22%);	A maioria leu 0 a 4 artigos da ASHA por ano sobre tópicos de avaliação (84%) ou intervenção (71%);	Demonstram grande interesse em treinamentos e recursos extras para apoiar práticas baseadas em evidências;	Um quarto dos respondentes não tinha treinamento formal em PBE;
	A estratégia PICO para formular perguntas clínicas foi mencionada como uma habilidade relevante.	O uso de recursos online da ASHA e o engajamento em atividades de PBE foram documentados como baixos;	96% estavam confiantes em sua capacidade de ler e compreender pesquisas;	91% não tinham tempo agendado para atividades de PBE;
		Pesquisas anteriores mostraram que as doses de tratamento utilizadas em estudos são geralmente maiores do que as viáveis na prática clínica.	88% reconheciam a importância de se manterem atualizados;	11% trabalhavam em distritos escolares com diretrizes oficiais de PBE;
			92% confiavam nos achados da maioria das pesquisas publicadas.	60% relataram não haver grupos de estudo de PBE formalmente organizados em seu distrito.
Mansuri, B. et al.^(5)^, 2020, Irã	Não possuíam conhecimento abrangente sobre a PBE;	O uso de evidências internas foi mais comum do que as evidências externas na prática clínica;	Apresentaram atitudes positivas e favoráveis em relação à PBE;	Não relatado.
	A pontuação média de conhecimento foi de 3.85 em uma escala de 0 a 10;	Evidências internas frequentemente usadas incluem experiência clínica própria (69,3%);	Houve alta concordância com as afirmações: "PBE é a base do desempenho profissional" (89,8%), "Tenho interesse em usar PBE na prática clínica" (97,3%) e "Preciso usar tratamentos baseados em evidências" (91,2%);	
	Fonoaudiólogos com pós-graduação obtiveram pontuações mais altas (média de 5.52) do que aqueles com graduação (média de 2.19);	Necessidades e preferências do cliente (84,2%)	Houve altas taxas de discordância com as afirmações: "PBE era uma perda de tempo" (93,6%) e "PBE era uma moda transitória" (79,5%).	
	Sugere-se que os fonoaudiólogos não receberam treinamento adequado em PBE em universidades ou programas educacionais.	Opiniões de colegas (57,2%) e consulta com especialistas (55,5%).		
		Entre as evidências externas, livros didáticos são usados por mais de 70%, recursos online como vídeos, áudios e internet por quase 40%, e grupos de Telegram/WhatsApp por 53,4%;		
		artigos científicos são menos utilizados em comparação.		
McCurtin, A.^(44),^ 2015, Irlanda	Qualificações adicionais e experiência clínica levam a tratamentos mais autônomos e científicos;	Decisões de tratamento baseiam-se principalmente na evidência da prática e em considerações pragmáticas;	Definiram como praticantes dinâmicos e pragmáticos com uma apreciação pelos quatro pilares da PBE;	Há escassez de evidências de pesquisa de alta qualidade para orientar decisões;
	Menor qualificação está ligada ao uso maior de produtos comerciais em vez de terapias baseadas em evidências;	3% relataram que a pesquisa não influencia suas decisões;	Os fatores que influenciam a tomada de decisões não se alinham claramente com os quatro pilares da PBE;	Clínicos menos experientes têm dificuldade em aplicar a pesquisa na prática;
	Maior experiência correlacionou-se com maior influência da própria experiência clínica.	A experiência clínica e a dos colegas, bem como a formação, influenciam as decisões.	58% concordaram que a ciência deve ser usada para decidir se as terapias funcionam;	45% dos respondentes sentem falta de tempo no trabalho para aprimorar tratamentos;
			48% concordaram que a experiência clínica é o melhor guia;	Para 39% dos fonoaudiólogos, a limitação de terapias e técnicas disponíveis é uma barreira.
			55% concordaram que podem desperdiçar tempo com tratamentos não validados, e 47% concordaram que podem causar danos;	
			4% acreditavam que qualquer tratamento funcionaria se o terapeuta acreditasse nele.	
Muttiah, N. et al.^(45)^, 2011, EUA	Apenas um clínico forneceu uma definição de PBE que incluía todos os três aspectos, as outras definições dos clínicos tendiam a focar em apenas um desses aspectos;	Decisões clínicas são guiadas principalmente pela experiência pessoal e opiniões de colegas, com menor uso da literatura científica. Os clínicos tendem a confiar mais em sua própria expertise do que nos demais pilares da PBE;	Ambos concordaram que a PBE é útil; clínicos a veem como um recurso para tornar o tratamento mais ético e credível, enquanto pesquisadores valorizam principalmente a evidência científica;	Tempo profissional foi a barreira mais mencionada para o uso da PBE, citada por metade dos respondentes;
	Alguns clínicos não estavam familiarizados com o uso da PBE ou se sentiam desconfortáveis com o termo.	85% relatou ter usado TONS nos últimos 5 anos, apesar da falta de evidência de pesquisa;	Há divergência sobre a responsabilidade pela eficácia: pesquisadores evitam o uso sem evidência, enquanto alguns clínicos aceitam o uso se não houver danos, o que contraria o Código de Ética da ASHA;	Até 22% dos respondentes apontaram como barreiras a quantidade e qualidade da pesquisa, recursos de busca e falta de conhecimento;
		Afirmaram que usariam a PBE apenas se ela apresentasse resultados, caso contrário, voltariam aos métodos anteriores.	Ambos concordaram que a pesquisa deve apoiar, mas não impor, as decisões clínicas.	Clínicos relataram dificuldade em acessar artigos devido à carga de trabalho e tempo limitado;
				A falta de evidência sobre os TONS frustrou ambos os grupos, atribuída à escassez de pesquisa em fonologia infantil e ao alto custo de estudos de eficácia.
Nail-Chiwetalu et al.^(46)^, 2007, EUA	66% interpretou a PBE como basear práticas na literatura de pesquisa;	Para questões clínicas, recorriam principalmente a colegas, educação continuada e pesquisas na internet aberta;	Consideraram mais úteis, para suas necessidades profissionais, as atividades de educação continuada, os contatos pessoais e a internet aberta;	A principal barreira relatada foi a falta de tempo para pesquisar e ler a literatura, representando 69% das barreiras;
	Apenas uma pequena parcela relatou ter recebido instrução em literacia da informação por bibliotecários, tanto formal (9%) quanto informalmente (5%);	Poucos utilizavam artigos acadêmicos para auxiliar em casos clínicos;	A PBE é vista como a integração da evidência de pesquisa com a expertise do profissional e os valores do cliente.	Outras barreiras incluíram dificuldade em localizar informações adequadas, uso de técnicas de busca e acesso a artigos em texto completo.
	60% se sentissem preparados pela pós-graduação para o aprendizado contínuo, suas respostas enfatizaram conteúdo, não habilidades de busca e avaliação da informação.	67% tenham consultado websites para informações clínicas, 59% não os acessavam rotineiramente para fins profissionais, enquanto 41% o faziam;		
		55% sentia-se muito bem-sucedida em analisar e aplicar as informações encontradas.		
O'Connor, S. et al.^(24)^, 2009, Irlanda	46,9% relataram que a análise estatística não é compreensível;	Há uma cultura predominante de uso de métodos tradicionais, em detrimento de abordagens baseadas em evidências;	A maioria dos terapeutas considerava a PBE essencial para a prática fonoaudiológica;	71,9% citaram falta de tempo para ler pesquisas como a principal barreira, enquanto 59,4% mencionaram falta de tempo para implementar novas ideias.
	37,5% não se sentiam capazes de avaliar a qualidade da pesquisa;	Diante de questões clínicas, profissionais tendem a se basear na experiência, opiniões de colegas, textos antigos ou websites generalizados, em vez da literatura atual;	6,3% não viam valor na pesquisa para a prática;	12,5% dos respondentes ou seus departamentos não assinavam revistas;
	Terapeutas de nível básico apresentaram maior dificuldade em compreender resultados estatísticos (72,8%) em comparação a terapeutas seniores (42,9%), indicando uma possível lacuna na formação ou na experiência.	Muitos fonoaudiólogos buscam novas práticas em cursos de desenvolvimento profissional e conversas com colegas experientes, em vez de artigos científicos.	62,5% apontaram inadequações metodológicas na pesquisa;	Foi relatada falta de pesquisa em áreas específicas da fonoaudiologia.
			53,1% acharam as implicações pouco claras;	
			Metade sentia que os resultados não eram aplicáveis ao seu contexto de trabalho;	
			15,6% relataram falta de apoio da equipe à implementação da PBE.	
Rojas, C. et al.^(47)^, 2023, América Latina	A maioria dos entrevistados entende a PBE apenas como publicações científicas, ignorando experiência e preferências dos usuários;	As respostas sobre o uso da PBE na tomada de decisões terapêuticas são variadas, com muitos limitando seu uso a áreas específicas, como disfagia, e priorizando a experiência profissional;	A maioria dos entrevistados acredita que a PBE se limita a publicações científicas;	A principal barreira é a falta de tempo para pesquisar e ler literatura científica atualizada;
	Essa visão é incorreta, já que a ASHA define a PBE como a combinação de pesquisa, experiência clínica e preferências do paciente;	Decisões são fundamentadas na experiência do profissional, práticas tradicionais e orientações dos ministérios da saúde.	Nenhum participante considera a PBE totalmente viável em seu contexto de trabalho, apesar de reconhecer sua importância;	A implementação é dificultada por contextos desfavoráveis, sessões curtas, acesso limitado a informações e barreiras linguísticas;
	É preciso aprimorar a formação dos fonoaudiólogos latino-americanos desde a graduação para corrigir esse equívoco.		Embora reconheçam a importância da PBE, há resistência à sua implementação prática.;	Falta de equipamentos, desconhecimento sobre busca científica, pouco engajamento dos profissionais e organizações inflexíveis;
			Existe a percepção de que propostas internacionais são desconectadas da realidade regional.	A crença na ausência de evidências válidas em algumas áreas reforça a continuidade de práticas tradicionais.
Sandham, V^(48)^. et al, 2021, Austrália	A falta de habilidade ou recursos para fornecer PBE foi relatada por 71% dos participantes;	87% discutem evidências com colegas para facilitar a PBE;	Os fonoaudiólogos mantêm a PBE em alta consideração, mas o estudo sugere que pode haver mais progresso em seu uso efetivo.	71% relataram falta de habilidade ou recursos para fornecer PBE;
	Fonoaudiólogos com pouca experiência na busca de literatura podem ter dificuldade significativa em encontrar e avaliar evidências externas.	75% conversam com clientes sobre evidências e preferências;		51% citaram recursos inadequados para implementar evidências de pesquisa como a principal barreira;
		64% leem revisões e participam de eventos;		36% mencionaram conflito entre evidências, valores do cliente e experiência clínica;
		61% consultam diretrizes profissionais;		26% identificaram barreiras organizacionais;
		68% aplicam medidas de resultado para avaliar sua prática e facilitar a PBE.		11% relataram falta de consenso entre colegas sobre a implementação da evidência;
				A falta de tempo foi destacada como um fator integral na implementação da PBE.
Sandham, V. et al.^(49)^, 2022, Austrália	A capacidade do clínico em PBE é essencial para oferecer serviços baseados em evidências;	Os clínicos buscam evidências focadas nas necessidades individuais dos clientes;	Os participantes sentem-se desconfortáveis ao usar evidências de qualidade duvidosa ou desconhecida;	Falta de recursos, tempo e custo dificultam o acesso e uso de evidências científicas;
	71% dos participantes relataram falta de habilidade ou recursos para fornecer PBE;	Participam de eventos educacionais e recorrem a colegas e mídias sociais para obter informações rápidas e acessíveis.	A falta de tempo para atividades de PBE está ligada à habilidade de pesquisa e crenças sobre seus benefícios;	Baixa confiança nas habilidades de busca e insegurança clínica são barreiras internas;
	Fonoaudiólogos com pouca experiência em busca de literatura enfrentam dificuldades para encontrar e avaliar evidências externas;		Há percepção de lacunas na pesquisa, especialmente para subpopulações específicas;	O desconforto com autorreflexão pode impedir o uso da PBE.
	A percepção da acessibilidade da evidência depende da habilidade de pesquisa e da experiência clínica para interpretar e aplicar os artigos.		Alguns consideram a pesquisa pouco aplicável ou de baixo valor clínico;	
			A experiência negativa ao acessar evidências reduz a motivação para buscar pesquisas originais.	
Souza et al.^(1)^, 2024, Brasil	56,6% indicaram ter aprendido as bases da PBE durante a formação acadêmica;	82% dos participantes frequentam congressos, cursos ou atualizações científicas (40,2% muito frequentemente; 41,8% frequentemente);	92,6% consideram a aplicação da PBE necessária na prática fonoaudiológica;	27,9% indicaram que o tempo disponível é insuficiente para executar a PBE;
	Há fragilidades no conhecimento e na habilidade de buscar e avaliar criticamente artigos científicos;	63,1% têm o hábito de ler artigos científicos (20,5% muito frequentemente; 42,6% frequentemente);	78,7% acreditam precisar aumentar o uso de evidências científicas no dia a dia;	55,8% concordam que faltam artigos que permitam generalizar os achados para a população de pacientes;
	Fonoaudiólogos com baixo domínio do inglês têm menos conhecimento e habilidades em PBE;	As bases de dados mais usadas são Scielo (33,6%), PubMed (24,8%) e Google Acadêmico (23,2%);	A maioria tem dúvidas ou discorda que a PBE traga retorno financeiro positivo;	56,5% reconhecem dificuldade em aplicar resultados científicos a pacientes com características únicas;
	A maioria discorda ou está indecisa sobre ter recebido treinamento formal em avaliação crítica (54,9%) e apenas 24,6% possuem treinamento em estratégias de busca;	Nenhum participante usa a base SpeechBite, e o acesso a Cochrane e ao mapa de evidências da ASHA é raro.	Fonoaudiólogos com baixo domínio do inglês tendem a achar que a PBE sobrecarrega o profissional (p=0,009);	48,4% apontam falta de apoio coletivo entre colegas para realizar a PBE;
	31,1% se sentem capazes de compreender análise estatística, enquanto outros 31,1% estão indecisos.		Profissionais com mais de 10 anos de formação concordam mais frequentemente sobre a falta de evidências para as intervenções usadas (p=0,046).	Fonoaudiólogos formados há menos de nove anos veem o gerenciamento do tempo para PBE como uma barreira maior (p=0,036);
				A área de linguagem infantil ainda carece de estudos científicos para intervenção.
Spek et al.^(50)^, 2013, Países Baixos	Estudantes mais avançados apresentaram pontuações significativamente maiores em conhecimento e habilidades em PBE;	Não relatado.	Estudantes de todos os anos valorizam positivamente a PBE, mas apresentam baixa autoeficácia em relação a suas competências;	A falta de sentimento de competência é uma barreira importante para o uso da PBE por estudantes;
	Todos os grupos avaliaram suas habilidades de busca como inadequadas, embora estudantes do 2º e 3º ano tenham pontuado essas habilidades como adequadas ou boas no DMF.		À medida que avançam nos estudos, mais estudantes percebem a PBE como demorada e sentem-se inseguros quanto à própria competência;	À medida que progridem no curso, mais estudantes consideram a PBE demorada, e isso desmotiva seu uso;
			Apesar disso, estudantes do terceiro ano consideram a PBE mais estimulante, com pontuações positivas para o valor da tarefa, ainda que as de autoeficácia permaneçam baixas.	Barreiras adicionais incluem: dificuldade com o inglês na leitura de artigos, escassez de evidências específicas na fonoaudiologia, dificuldade para validar essas evidências clinicamente, acesso limitado a bases de dados e a "numerofobia" (medo de números) em alunos sem formação anterior em matemática.
Thome et al.^(51)^, 2018, EUA	13% identificaram corretamente os três componentes da PBE;	52% acessam recursos da ASHA para informações profissionais;	99% consideram a PBE benéfica para oferecer melhores tratamentos;	51% apontaram a falta de tempo para pesquisar e ler como a principal barreira à PBE;
	35% classificaram a meta-análise como o nível mais forte de evidência;	21% recorrem a contatos pessoais como segunda fonte mais frequente;	99% acham importante engajar-se na PBE;	27% citaram acesso limitado à informação;
	36% indicaram os ensaios clínicos randomizados como segundo nível de evidência;	29% consideram bancos de dados online não via bibliotecas como fontes improváveis de acesso;	62% acham difícil se engajar na PBE;	24% mencionaram a falta de informações relevantes para seus casos clínicos;
	61% classificaram corretamente relatos de especialistas e experiências clínicas como o nível mais fraco.	Bibliotecas universitárias/públicas/médicas (35%) e websites não acadêmicos (43%) são os menos acessados;	A maioria considera que seus empregadores conhecem a PBE (33% “muito conhecedor”, 45% “um tanto conhecedor”) e incentivam seu uso (43% “concordo totalmente”, 36% “concordo um pouco”);	Apenas 15% disseram que seu empregador fornece tempo para atualização em PBE;
	23% se sentem "muito conhecedores" no uso de bibliotecas acadêmicas e médicas; 35% disseram o mesmo sobre bases de dados online fora das bibliotecas;	O estudo destaca que nem sempre o uso autodeclarado da PBE significa aderência real às diretrizes da PBE.	Fontes mais úteis: recursos da ASHA (46%) e contatos pessoais (27%);	Apenas 24% afirmaram que seu empregador oferece acesso a conteúdos pagos (bancos de dados ou periódicos).
	49% se sentem "muito confiantes" e 45% "um tanto confiantes" em ler e interpretar estudos científicos.		Fontes menos úteis: websites não acadêmicos (35%) e bibliotecas universitárias/públicas/médicas (36%).	
Tohidast et al.^(52)^, 2017, Irã	46,4% recebeu educação formal sobre PBE na universidade, e cerca de metade não teve esse tipo de formação;	53% leram entre 2 e 5 artigos no último mês; apenas 3,2% leram mais de 15;	Os fonoaudiólogos iranianos tinham uma atitude positiva em relação à PBE;	62% apontaram a falta de tempo como a principal barreira para implementar a PBE;
	63% relatou confiança em suas habilidades de busca, 67% avaliação crítica, embora muitos não compreendam termos técnicos;	38,6% pesquisaram em bancos de dados menos de 2 vezes no mês;	Os fonoaudiólogos concordaram ou concordaram fortemente que a PBE é necessária para a fonoaudiologia (96,8%) e que aumenta a qualidade do cuidado ao paciente (96,1%).	44,7% relataram falta de habilidades de pesquisa e 40,7% destacaram a dificuldade de aplicar resultados da pesquisa a pacientes com características únicas;
	Treinamento formal aumentou a confiança nas habilidades em PBE, sendo que mulheres relataram mais confiança do que homens para implementá-la.	81% usaram literatura para decisões clínicas menos de 6 vezes no mês;	81,4% dos respondentes discordaram ou discordaram fortemente de que a adoção da PBE impõe uma demanda irracional aos profissionais;	A falta de interesse foi considerada a barreira menos relevante.
		52% tinham acesso a periódicos impressos;	73,6% discordaram ou discordaram fortemente de que a PBE não considera as preferências do paciente.	
		45,7% tinham acesso a bancos de dados no trabalho, enquanto 87% tinham acesso em casa ou outro local.		
Tohidast et al.^(53)^, 2021, Irã	Não relatado.	Não relatado.	Algumas atitudes pessoais negativas funcionam como barreiras, como sentir-se cientificamente competente demais ou achar que o conhecimento atual é suficiente, sem buscar atualização;	Falta de conhecimento e habilidades em PBE;
			O orgulho excessivo leva à crença de que técnicas e métodos atuais bastam, sem necessidade de aprender novos;	Atitudes pessoais negativas (excesso de confiança no conhecimento atual, resistência à aprendizagem)
			A cultura social influencia as opiniões, incluindo uma "cultura do conforto" que prefere formas passivas de aprendizado, como palestras e workshops, evitando esforço próprio.	Falta de tempo para pesquisar e aplicar a PBE;
				Educação acadêmica insuficiente sobre PBE e pesquisa científica;
			Existe uma cultura centrada no médico/clínico, onde pacientes e famílias confiam demais nas decisões do profissional e evitam participar ativamente nas decisões clínicas, contrariando princípios da PBE.	Infraestrutura inadequada nas clínicas para implementação da PBE.;
				Pressões financeiras para prescrever mais sessões, contrariando a PBE;
				Escassez de evidências e diretrizes específicas para o contexto regional;
				Baixa conscientização pública sobre a PBE, gerando resistência familiar;
				Limitações financeiras das famílias que dificultam o acesso a tratamentos baseados em evidências.
Vallino-Napoli, L. D. et al.^(54)^, 2004, Austrália	A maioria dos clínicos conhecia a PBE e tinha uma noção do seu significado;	Todos os respondentes participaram de educação continuada no último ano e tinham acesso à Internet, mas acessavam essas fontes com pouca frequência;	A maioria dos clínicos havia ouvido falar de PBE e valorizava muito a pesquisa;	A falta de tempo para leitura e pesquisa é a principal barreira para a PBE;
	94% associavam PBE à aplicação de resultados de estudos clinicamente relevantes;	53% dos clínicos usaram bancos de dados de computador para pesquisa de literatura;	75% concordaram que "Todos os fonoaudiólogos deveriam fazer um curso obrigatório sobre metodologia de pesquisa";	93% concordam que deveria haver tempo alocado para isso, mas 69% relataram não ter tempo dedicado no trabalho;
	51% incluíam a aplicação de habilidades e experiência clínica na definição de PBE;	90% usaram periódicos profissionais como fonte de evidência;	66% concordaram que "Pesquisa me ajuda a alcançar o que quero como fonoaudiólogo";	A frequência limitada de acesso às fontes de evidência é a segunda barreira;
	28% consideravam importante integrar as opiniões dos pacientes nas decisões clínicas;	72% dependiam de diretrizes clínicas estabelecidas;	44% concordou que "É difícil aplicar a pesquisa na prática"	Dificuldade em compreender estatísticas nos estudos (78% em estudo citado);
	3% não sabiam o que PBE significava;	Fontes mais procuradas para manejo de pacientes: palestras (89%), consulta com especialista local (86%), artigos de periódicos (70%), Internet (64%), grupos de interesse especial (53%), clubes de leitura de periódicos (51%).	26% concordaram que "Basear a prática em achados de pesquisa custaria muito tempo e dinheiro";	Implicações para a prática pouco claras (66% no estudo citado);
	Clínicos com até 10 anos de prática usavam mais frequentemente achados de pesquisa para guiar a prática, possivelmente devido a mudanças no currículo.		14% concordaram que "A maioria dos fonoaudiólogos não está interessada em implementar achados de pesquisa".	Literatura dispersa em muitos periódicos;
				Acesso a recursos computacionais pode ser demorado e ineficiente, o que desestimula clínicos ocupados.
Zipoli Jr, R. P. et al.^(25)^, 2005, EUA	13% dos fonoaudiólogos relataram falta de conhecimento e habilidades como barreira à PBE, indicando que a maioria se sente competente;	Fontes mais usadas: experiência clínica própria (99,6%) e opiniões de colegas (78,7%);	Os fonoaudiólogos demonstraram atitudes geralmente positivas em relação à pesquisa e à PBE;	A falta de tempo profissional foi a barreira mais significativa, com 50,0% dos respondentes discordando ou discordando fortemente que tinham tempo para participar da PBE;
	A formação de pós-graduação, que pode incluir treinamento em estatística e métodos de pesquisa, contribui para essa competência;	Fontes menos usadas: estudos de caso (15,9%), vídeos ou audiotapes (17,3%) e estudos de pesquisa (17,7%);	Foi encontrada uma correlação positiva modesta (r = 0,438, p < 0,001) entre essas atitudes e o uso auto-relatado da PBE.	Barreiras relacionadas a conhecimento e habilidades (13,0%), recursos (17,6%) e quantidade/qualidade da pesquisa (21,8%) foram percebidas em menor grau pelos fonoaudiólogos deste estudo.
	A exposição à pesquisa e à PBE durante a pós-graduação e o CFY é um preditor importante de atitudes positivas em relação à PBE.	Houve uma queda significativa na exposição à pesquisa e PBE do período de pós-graduação (média 2,19) para o CFY (média 3,23), sendo que valores mais altos indicam menor exposição.		

**Legenda:** PBE: Prática Baseada em evidências; TEA: Transtorno do espectro autista; ASHA: American Speech-Language-Hearing Association; CAS: Childhood Apraxia of Speech; TONS: Tratamentos Oromotores Não-Fonoarticulatórios; CFY: Ano de Estágio Clínico
